# Mapping the Interface of a GPCR Dimer: A Structural Model of the A_2A_ Adenosine and D_2_ Dopamine Receptor Heteromer

**DOI:** 10.3389/fphar.2018.00829

**Published:** 2018-08-30

**Authors:** Dasiel O. Borroto-Escuela, David Rodriguez, Wilber Romero-Fernandez, Jon Kapla, Mariama Jaiteh, Anirudh Ranganathan, Tzvetana Lazarova, Kjell Fuxe, Jens Carlsson

**Affiliations:** ^1^Department of Neuroscience, Karolinska Institute, Stockholm, Sweden; ^2^Science for Life Laboratory, Department of Biochemistry and Biophysics, Stockholm University, Stockholm, Sweden; ^3^Science for Life Laboratory, Department of Cell and Molecular Biology, Uppsala University, Uppsala, Sweden; ^4^Department of Biochemistry and Molecular Biology, Institute of Neuroscience, Faculty of Medicine, Autonomous University of Barcelona, Barcelona, Spain

**Keywords:** G protein-coupled receptor, D_2_ dopamine receptor, A_2A_ adenosine receptor, heteroreceptor complex, dimerization, dimer interface, allosteric modulation

## Abstract

The A_2A_ adenosine (A_2A_R) and D_2_ dopamine (D_2_R) receptors form oligomers in the cell membrane and allosteric interactions across the A_2A_R–D_2_R heteromer represent a target for development of drugs against central nervous system disorders. However, understanding of the molecular determinants of A_2A_R–D_2_R heteromerization and the allosteric antagonistic interactions between the receptor protomers is still limited. In this work, a structural model of the A_2A_R–D_2_R heterodimer was generated using a combined experimental and computational approach. Regions involved in the heteromer interface were modeled based on the effects of peptides derived from the transmembrane (TM) helices on A_2A_R–D_2_R receptor–receptor interactions in bioluminescence resonance energy transfer (BRET) and proximity ligation assays. Peptides corresponding to TM-IV and TM-V of the A_2A_R blocked heterodimer interactions and disrupted the allosteric effect of A_2A_R activation on D_2_R agonist binding. Protein–protein docking was used to construct a model of the A_2A_R–D_2_R heterodimer with a TM-IV/V interface, which was refined using molecular dynamics simulations. Mutations in the predicted interface reduced A_2A_R–D_2_R interactions in BRET experiments and altered the allosteric modulation. The heterodimer model provided insights into the structural basis of allosteric modulation and the technique developed to characterize the A_2A_R–D_2_R interface can be extended to study the many other G protein-coupled receptors that engage in heteroreceptor complexes.

## Introduction

G protein-coupled receptors (GPCRs) play essential roles in physiological processes and are important therapeutic targets (Lefkowitz, 2004; Lagerstrom and Schioth, 2008). The vast majority of the research efforts that have been made to understand GPCR signaling was performed under the assumption that these membrane proteins function as monomers. Contrary to this view, there is now evidence suggesting that many GPCRs form homo- and heteromers at the cell surface (Franco et al., 2000; Overton and Blumer, 2000; Angers et al., 2001; Han et al., 2009; Hasbi et al., 2009, 2017; Navarro et al., 2010; Vidi et al., 2010; Borroto-Escuela et al., 2014; Fuxe et al., 2014; Meng et al., 2014; Marsango et al., 2015; Martinez-Munoz et al., 2016; Rico et al., 2017). At the molecular level, GPCR signaling is hence not only determined by conformational changes induced by agonist binding, but is also allosterically modulated by interactions with other receptors. Further characterization of the influence of receptor–receptor interactions on signaling will be crucial for understanding GPCR function and could lead to development of novel drugs.

A prototypical GPCR heteromer is the one formed by the A_2A_ adenosine receptors (A_2A_R) and D_2_ dopamine receptors (D_2_R) (Fuxe et al., 2010). *In vitro* experiments demonstrated that activation of the A_2A_R reduces D_2_R high affinity binding of agonists, suggesting that allosteric receptor–receptor interactions in the plasma membrane influence D_2_R signaling (Ferre et al., 1991). This was further supported by the demonstration that the A_2A_R and D_2_R form heteroreceptor complexes with antagonistic receptor–receptor interactions in living cells and in brain tissue (Hillion et al., 2002; Fuxe et al., 2005). A considerable amount of experimental data, including biophysical, biochemical, chemical neuroanatomical, and behavioral studies support the view that A_2A_R–D_2_R heteromerization plays an important functional role in the basal ganglia (Ferre et al., 1991; Yang et al., 1995; Dasgupta et al., 1996; Fenu et al., 1997; Hillion et al., 2002; Canals et al., 2003; Kamiya et al., 2003; Ciruela et al., 2004; Fuxe et al., 2005, 2007b, 2010; Azdad et al., 2009; Borroto-Escuela et al., 2010a,b, 2018; Navarro et al., 2010; Fernandez-Duenas et al., 2012; Cordomi et al., 2015; Feltmann et al., 2018). The negative allosteric modulation exerted by A_2A_R activation on D_2_R signaling provided one mechanism contributing to the motor activation observed with general adenosine receptor antagonists (e.g., caffeine) and the neuroleptic effects of A_2A_R agonists (Fuxe et al., 2007a). For this reason, the A_2A_R–D_2_R heteroreceptor complex represents a new therapeutic target for disorders treated with drugs interacting with the D_2_R, such as neurodegenerative diseases, schizophrenia, and drug addiction (Borroto-Escuela et al., 2016b). For example, A_2A_R antagonists should *inter alia* enhance dopaminergic signaling by blocking A_2A_R activation by endogenous adenosine in A_2A_R–D_2_R oligomers, thereby reducing the negative allosteric modulation of the D_2_R protomer (Fuxe et al., 2015). This may contribute to the antiparkinsonian effects observed after treatment with A_2A_R antagonists, such as reduction of bradykinesia, motor fluctuations, and dyskinesia associated with chronic L-DOPA treatment (Schwarzschild et al., 2006; Armentero et al., 2011).

Despite the thorough characterization of GPCR heteromers by biophysical and biochemical methods (Angers et al., 2001; Vidi et al., 2010; Fernandez-Duenas et al., 2012; Borroto-Escuela et al., 2013; Fuxe et al., 2014; Marsango et al., 2015; Fuxe and Borroto-Escuela, 2016), the understanding of the structural basis of GPCR heteromerization and the associated negative and positive cooperativity remains limited. A major advancement in this area was the recent determination of GPCR crystal structures, which enable generation of atomic resolution models for homo- and heteroreceptor complexes. Crystal structures for representative members from the adenosine (Liu et al., 2012) and dopamine (Chien et al., 2010; Wang et al., 2018) receptor families have recently been solved. In addition, a number of class A GPCRs have crystallized as homodimers (Murakami and Kouyama, 2008; Wu et al., 2010; Granier et al., 2012; Manglik et al., 2012; Huang et al., 2013), providing different plausible orientations of two monomers relative to each other. The rapidly increasing amount of structural data can now contribute to generation of models of GPCR dimers, which could improve understanding of allosteric modulation.

In this work, a combined computational and experimental approach to construct atomic resolution models of GPCR dimers was developed. In order to predict the structure of the A_2A_R–D_2_R heterodimer, regions involved in the interface were identified based on experiments utilizing peptides corresponding to the transmembrane (TM) helices of the A_2A_R and D_2_R. The effects of synthetic peptides corresponding to the seven TM helices of the A_2A_R on A_2A_R–D_2_R heteromerization were assessed using bioluminescence resonance energy transfer (BRET) (Borroto-Escuela et al., 2013) and proximity ligation assays (PLAs). Combined with the corresponding BRET data for the seven TM peptides derived from the D_2_R sequence (Borroto-Escuela et al., 2010b), an initial model of the A_2A_R–D_2_R heteromer was constructed computationally using protein–protein docking. The predicted heterodimer structure was refined using molecular dynamics (MD) simulations. The resulting A_2A_R–D_2_R structure was subsequently used to predict mutations in the dimer interface, which were evaluated experimentally to assess the model.

## Materials and Methods

### Plasmid Constructs

The cDNA encoding the human D_2_R, human A_2A_R, and human A_1_R cloned in pcDNA3.1+ were subcloned (without stop codons) in humanized pGFP^2^-N1 (PerkinElmer, Waltham, MA, United States), pEYFP-N1 (Clontech, Germany), and p*R*luc-N3 vectors (Packard Bioscience, Spain). The cDNA encoding human 3xHA-D_2_R cloned in pcDNA3.1+ and human D_2_R cloned in pGFP^2^-N1 (Perkin-Elmer, Spain) were used as template to generate the two mutants, Tyr192Ala^5.41x42^ and Leu207Ala^5.56^/Lys211Ala^5.60^ [generic GPCR residue numbers indicated in superscript (Isberg et al., 2015)], by means of the QuickChange^TM^ site-directed mutagenesis kit (Stratagene, Netherlands) following the manufacturer’s protocol. The other constructs used have been described previously (Borroto-Escuela et al., 2010a,b).

### Cell Culture and Transfection

HEK293T cells (American Type Culture Collection, United States) were grown in Dulbecco’s modified Eagle’s medium supplemented with 2 mM L-glutamine, 100 units/ml penicillin/streptomycin, and 10% (v/v) fetal bovine serum (FBS) at 37°C in an atmosphere of 5% CO_2_. Cells were plated in 6-well plates at a concentration of 1 × 10^6^ cells/well or in 75 cm^2^ flasks and cultured overnight prior to transfection. Cells were transiently transfected using linear polyethylenimine (Polysciences Inc., United States). Rat primary striatal neuronal cells purchased from QBM Cell Science (Montreal, QC, Canada) were cultured in Neuro basal medium supplemented with 10% FBS, 2 mM GlutaMAX-1, 1 mM sodium pyruvate, 100 U/ml penicillin G, and 100 μg/ml streptomycin and B-27 supplement at 37°C in a humidified 10% CO_2_ environment according to manufacturer’s instructions.

### TM Peptide Treatment

Synthetic peptides, representing each of the TM peptides for the human A_2A_R, were obtained from Lazarova et al. (2004), Thevenin et al. (2005), Thevenin and Lazarova (2008), CASLO (Denmark) or Ana Spec Inc. (CA, United States) with ≥90% purity. The A_2A_R TM-I peptide consisted of residues 8–32 (VYITVELAIAVLAILGNVLVCWAVW); TM-II peptide of residues 43–66 (YFVVSLAAADIAVGVLAIPFAITI); TM-III peptide of residues 78–100 (LFIACFVLVLTQSSIFSLLAIAI); TM-IV peptide of residues 121–143 (AKGIIAICWVLSFAIGLTPMLGW); TM-V peptide of residues 174–198 (MNYMVYFNFFACVLVPLLLMLGVYL); TM-VI peptide of residues 235–261 (LAIIVGLFALAWLPLHIINCFTFFAPD); and TM-VII peptide of residues 267–291 (LWLMYLAIVLSHTNSVVNPFIYAYR). A di, tri- or quatribasic sequence (KK, KKK, or RKKK) was introduced at the N- and C-terminal to ensure incorporation into the plasma membrane of cells, as demonstrated previously (Borroto-Escuela et al., 2012, 2018). The synthetic peptide representing TM-V of the 5-HT_1A_ receptor was described previously (Borroto-Escuela et al., 2012, 2015a,b). Immediately before use, the peptides were solubilized in dimethyl sulfoxide (DMSO) and diluted in the corresponding cell culture medium to yield a final concentration of 1% DMSO. We verified that, for each tested concentration of DMSO alone, no effect on cell viability was observed. Cells were incubated with the above mentioned peptides at 37°C for 2 h prior to performing BRET analysis, *in situ* PLA assays or binding assays.

### BRET^1^ Assays

In the BRET^1^ assays, the receptors of interest were fused to either Renilla luciferase (RLuc) or yellow fluorescent protein (YFP). Detection of dimer interactions is based on that RLuc oxidation of the substrate coelenterazine *h* leads to light emission with a peak at 480 nm, which results in excitation of YFP (excitation and emission maxima of 475 and 530 nm, respectively) if the receptors are in close proximity (Pfleger and Eidne, 2006). In the BRET^1^ saturation assays (Borroto-Escuela et al., 2013), HEK293T cells were transiently co-transfected with constant amounts (1 μg) of plasmids encoding for D_2_R^Rluc^ and increasing amounts (0.5–8 μg) of plasmids encoding for A_2A_R^Y FP^. Forty-eight hours after transfection the cells were rapidly washed twice in PBS, detached, and resuspended in the same buffer. Cell suspensions (20 μg proteins) were distributed in duplicate into 96-well microplates; black plates with transparent bottom (Corning 3651, Corning, Stockholm, Sweden) for fluorescence measurement or white plates with white bottom (Corning 3600) for BRET determination. For BRET^1^ ratio measurement, coelenterazine *h* substrate (Molecular Probes, Eugene, OR, United States) was added at a final concentration of 5 μM. Readings were performed after 1 min and the BRET signal was detected using the POLARstar Optima plate reader (BMG Lab Technologies, Offenburg, Germany) that allows the sequential integration of the signals detected with two filter settings. Transfected HEK293T cells were incubated with 0.1 μM of the A_2A_R TM-V peptide at 37°C for 2 h prior to performing BRET^1^ analysis. Data were then represented as a normalized netBRET^1^ ratio versus the fluorescence value obtained from the YFP, normalized with the luminescence value of D_2_R^Rluc^ expression 10 min after h-coelenterazine incubation. The normalized netBRET^1^ ratio was defined as the BRET ratio for co-expressed Rluc and YFP constructs normalized against the BRET ratio for the Rluc expression construct alone in the same experiment: netBRET^1^ ratio = [(YFP emission at 530 ± 10 nm)/(Rluc emission 485 ± 10 nm)] – cf. The correction factor, cf. corresponds to (emission at 530 ± 10 nm)/(emission at 485 ± 10 nm) found with the receptor-Rluc construct expressed alone in the same experiment. BRET isotherms were fitted using a non-linear regression equation assuming a single binding site, which provided BRETmax and netBRETmax values. The maximal value of BRET (BRETmax or netBRETmax) corresponds to the situation when all available donor molecules are paired up with acceptor molecules.

To assess specificity of the peptides, HEK293T cells were transiently co-transfected with plasmids encoding for D_2_R^Rluc^ or A_2A_R^Rluc^ and A_2A_R^Y FP^ (pcDNA ratio 1:1, 1 μg cDNA each). Forty-eight hours after transfection the cells were incubated with 10 μM of the A_2A_R TM-IV peptide, or 0.1 μM of the A_2A_R TM-V peptide, or 0.1 μM of the 5-HT_1A_ TM-V peptide at 37°C for 2 h prior to performing BRET^1^ analysis. The netBRET signal was detected as described above.

In the BRET^1^ competition assays, HEK293T cells transiently co-transfected with constant amounts (1 μg) of plasmids encoding for D_2_R^Rluc^ and A_2A_R^Y FP^ were incubated with each of the seven A_2A_R TM peptides at 37°C for 2 h prior to performing BRET^1^ analysis. The netBRET signal was detected as described above.

### BRET^2^ Saturation Assays

In the BRET^2^ assays, the receptors of interest were fused to either RLuc or green fluorescent protein 2 (GFP2). Detection of dimer interactions is based on that Rluc oxidation of coelenterazine 400a (DeepBlueC) leads to light emission with a peak at 395 nm, which results in excitation of GFP^2^ (excitation and emission maxima of 400 and 510 nm, respectively) if the receptors are in close proximity (Pfleger and Eidne, 2006). The BRET^2^ saturation assays (Borroto-Escuela et al., 2013) were carried out using plasmids encoding for A_2A_R^Rluc^ and either 3xHA-D_2_R^GFP2^, 3xHA-D_2_R^GFP2^(Tyr192Ala^5.41x42^) or 3xHA-D_2_R^GFP2^(Leu207Ala^5.56^/Lys211Ala^5.60^), respectively. Forty-eight hours after transfection, HEK293T cells transiently transfected with constant (1 μg) or increasing amounts (0.12–7 μg) of plasmids encoding for A_2A_R^Rluc^ and either 3xHA-D_2_R^GFP2^, 3xHA-D_2_R^GFP2^(Tyr192Ala^5.41x42^) or 3xHA-D_2_R^GFP2^(Leu207Ala^5.56^/Lys211Ala^5.60^), respectively, were rapidly washed twice in PBS, detached, and resuspended in the same buffer. Cell suspensions (20 μg protein) were distributed in duplicate into the 96-well microplates; black plates with a transparent bottom (Corning 3651) (Corning, Stockholm, Sweden) for fluorescence measurement or white plates with a white bottom (Corning 3600) for BRET determination. For BRET^2^ measurement, DeepBlue*C* substrate (VWR, Sweden) was added at a final concentration of 5 μM, and readings were performed after 1 min using the POLARstar Optima plate-reader (BMG Lab Technologies, Offenburg, Germany) that allows the sequential integration of the signals detected with two filter settings. The netBRET^2^ ratio was defined as the BRET ratio for co-expressed Rluc and GFP^2^ constructs normalized against the BRET ratio for the Rluc expression construct alone: netBRET^2^ ratio = [(YFP emission at 515 ± 30 nm)/(Rluc emission 410 ± 80 nm)] – cf. The correction factor, cf, corresponds to (emission at 515 ± 30 nm)/(emission at 410 ± 80 nm) found with the receptor-Rluc construct expressed alone in the same experiment. The maximal value of BRET (BRET^2^max or netBRET^2^max) corresponds to the situation when all available donor molecules are paired up with acceptor molecules (Fuxe et al., 2010). The specificities of A_2A_R–D_2_R interactions were assessed by comparison with co-expression of A_1_R^Rluc^ and D_2_R^GFP2^.

### *In situ* Proximity Ligation Assay

HEK293T cells transiently co-transfected with constant amounts (1 μg) of plasmids encoding for A_2A_R and D_2_R and rat primary striatal neuronal cells were employed to study the effect of TM peptides on A_2A_R–D_2_R complexes by means of *in situ* PLA. Furthermore, to study the effect of D_2_R mutants on A_2A_R–D_2_R complexes by means of *in situ* PLA, HEK293T cells were also transiently co-transfected with constant amounts (1 μg) of plasmids encoding for A_2A_R and 3xHA-D_2_R or 3xHA-D_2_R(Tyr192Ala^5.41x42^) or 3xHA-D_2_R(Leu207Ala^5.56^/Lys211Ala^5.60^). *In situ* PLA was performed using rabbit polyclonal anti-A_2A_R (Abcam: ab3461 or Millipore: AB1559) and mouse monoclonal anti-D_2_R (Millipore MABN53, clone 3D9) primary antibodies (for quality control of the antibodies, see Feltmann et al., 2018) and the Duolink *in situ* PLA detection kit (Sigma-Aldrich, Sweden), following the protocol described previously (Borroto-Escuela et al., 2012, 2016a, 2018; Feltmann et al., 2018). Primary striatal neuronal cells or transiently co-transfected HEK293T cells were incubated with 10 μM of the A_2A_R TM-IV peptide or 0.1 μM of the A_2A_R TM-V peptide at 37°C for 2 h prior to performing cell fixation with 3.7% formaldehyde solution (Sigma-Aldrich, Stockholm, Sweden). PLA control experiments employed only one primary antibody or cells transfected with cDNAs encoding only one type of receptor. The PLA signal was visualized and quantified by using a TCS-SL confocal microscope (Leica, United States) and the Duolink Image Tool software. High magnifications of the microphotograph were taken and visualized using multiple z-scan projection. It should be noted that if the confocal data acquisition is performed as a multiple z-scan some positive PLA blobs/clusters may appear to be inside the nuclear blue stained DAPI area despite being located on the cytoplasmic membrane.

### Cell Surface Receptor Expression and Cellular Localization Analysis by Fluorescence Confocal Microscopy

HEK293T cells were transiently transfected with constant amounts (1 μg) of plasmids encoding for 3xHA-D_2_R^GFP2^, 3xHA-D_2_R^GFP2^(Tyr192Ala^5.41x42^) or 3xHA-D_2_R^GFP2^(Leu207Ala^5.56^/Lys211Ala^5.60^). Then, cells were fixed in 4% paraformaldehyde for 10 min, washed with PBS containing 20 mM glycine, and mounted in a Vectashield immunofluorescence medium (Vector Laboratories, United Kingdom). Microscope observations were performed with a 40× oil immersion objective in a Leica TCS-SL confocal microscope (Leica, United States).

### [^3^H]-Raclopride Competition Experiments

[^3^H]-Raclopride binding was displaced by quinpirole to determine the proportion of receptors in the high affinity state (RH), the high affinity (K*_i,High_*), and low affinity (K*_i,Low_*) values for the agonist binding sites from competition curves in HEK cells expressing either A_2A_R/3xHA-D_2_R, A_2A_R/3xHA-D_2_R(Tyr192Ala^5.41x42^), or A_2A_R/3xHA-D_2_R(Leu207Ala^5.56^/Lys211Ala^5.60^). Membrane preparations (60 μg protein/ml) were incubated with increasing concentrations of quinpirole (0.001 nM to 1 μM) and 2 nM [^3^H]-raclopride (75 Ci/mmol, Novandi Chemistry AB, Sweden) in 250 μl of incubation buffer (50 mM Tris-HCl, 100 mM NaCl, 7 mM MgCl_2_, 1 mM EDTA, 0.05% BSA, 1 mM DTT) and 0.3 IU/ml adenosine deaminase (EC 3.5.4.4, Sigma-Aldrich) for 90 min at 30°C in the presence or absence of 100 nM of the A_2A_R agonist CGS-21680. Non-specific binding was defined by radioligand binding in the presence of 10 μM (+) butaclamol (Sigma-Aldrich, Sweden). The incubation was terminated by rapid filtration Whatman GF/B filters (Millipore Corp, Sweden) using a MultiScreen^TM^ Vacuum Manifold 96-well followed by three washes (∼250 μl per wash) with ice-cold washing buffer (50 mM Tris-HCl pH 7.4). The filters were dried, 5 ml of scintillation cocktail was added, and the amount of bound ligand was determined after 12 h by liquid scintillation spectrometry.

### Statistical Analysis

The number of samples (*n*) in each experimental condition is indicated in figure legends. When two experimental conditions were compared, statistical analysis was performed using an unpaired *t*-test. Data from the competition experiments were analyzed by non-linear regression analysis. The changes induced by CGS-21680 on RH, K*_i,High_*, and K*_i,Low_* values were compared using one-way ANOVA. A *p*-value ≤ 0.05 was considered significant.

### Homology Modeling

An atomic resolution structure of the D_2_R was constructed using homology modeling based on a crystal structure of the D_3_ subtype as template (PDB code 3PBL) (Chien et al., 2010). A set of 100 homology models was generated with MODELLER v9.10 (Sali and Blundell, 1993) based on a manually edited sequence alignment (**Supplementary Figure S1**) generated by the GPCR-ModSim webserver (Rodríguez et al., 2012). The D_2_R residues Arg31^1.29^-Tyr36^1.34^ were not present in the corresponding region of the template and alpha-helical restraints were added for these to extend TM-I. In addition, 140 residues from the intracellular loop 3 (IL3) of the D_2_R were omitted by introducing a chain break between Arg222^5.71^ and Leu363^6.25^. The ends of helices TM-V and TM-VI, which are connected by the IL3, were treated as independent. Selection of a final homology model was based on the DOPE scoring function (Shen and Sali, 2006), visual inspection, and the metrics available from the Molprobity server (Davis et al., 2007).

### Protein–Protein Docking

A crystal structure of the A_2A_R (PDB code 4EIY) (Liu et al., 2012) and the D_2_R homology model were prepared for protein–protein docking with the program HADDOCK2.1 (Dominguez et al., 2003). The protonation states of ionizable residues (Asp, Glu, Arg, and Lys) were set to their most probable states at pH 7. Histidine protonation states were set by visual inspection on the basis of the hydrogen bonding network (**Supplementary Table S1**). Residues in the predicted dimer interface were used to guide protein–protein docking (**Supplementary Table S2**). A set of 1,000 configurations of the A_2A_R–D_2_R heterodimer was generated by rigid-body energy minimization. The 200 complexes with the best energy were refined and clustered by HADDOCK. Each A_2A_R–D_2_R complex was refined with MD sampling in the presence of explicit DMSO molecules to mimick the membrane environment. Subsequent to the determination of the first D_2_R crystal structure (PDB code 6CM4) (Wang et al., 2018), the protein–protein docking was repeated with the same parameters. In this case, 10,000 configurations of the A_2A_R–D_2_R dimer were generated and 1,000 of these were refined. The resulting models were clustered (Rodrigues et al., 2012) based on superimposition to the A_2A_R with a 7.5 Å RMSD cutoff and a minimum cluster size of four members.

### MD Simulations

A model of the A_2A_R–D_2_R complex was prepared for all-atom MD simulations in GROMACS4.5.5 (Pronk et al., 2013). Prior to the MD simulations, the stretch of residues surrounding Ser197^5.46x461^ in the extracellular tip of TM-V of the D_2_R was modified to preserve the alpha-helical secondary structure present in the D_3_R template, which had slightly unfolded in the HADDOCK optimization of the heteromer complex. The dimer was inserted into a hydrated 1-palmitoyl-2-oleoylphosphatidylcholine (POPC) bilayer in gel phase (equilibrated at 260 K). Water and lipid atoms overlapping with the protein were removed, and the simulation box walls were set to be 12 and 35 Å from the protein atoms in the Z and XY dimensions, respectively. A 0.15 M sodium concentration and neutralization of the system were accomplished by adding 100 sodium and 118 chloride ions to the system. The resulting hexagonal prism-shaped simulation box comprised 145,260 atoms, including 36,889 water and 438 lipid molecules, and had an initial volume of 156 × 156 × 99 Å^3^. MD simulations were performed using the half-e double-pairlist method (Chakrabarti et al., 2010) to make the Berger parameters employed for the lipids (Berger et al., 1997) compatible with the OPLS-AA force field (Jorgensen et al., 1996) used for the protein atoms. As a part of the IL3 of the D_2_R was excluded from modeling, C-terminal amide and N-terminal acetyl caps were added to avoid charge–charge interactions between the termini of residues Arg222 and Leu363, respectively. Similarly, the N-termini of both the A_2A_R (Gly5^1x31^) and D_2_R (Tyr34^1x32^) were capped. The system was solvated with SPC waters (Jorgensen et al., 1983). Bond lengths and angles for these molecules were constrained with the SETTLE algorithm (Miyamoto and Kollman, 1992), whereas the LINCS algorithm (Hess et al., 1997) was used to constrain bond lengths of proteins and lipids. Periodic-boundary conditions were applied in the NPT ensemble using the semiisotropic Parinello-Raman barostat (Parrinello and Rahman, 1981) (1 atm, coupling constant of 2 ps, isothermal compressibility constant of 4.5 × 10^-5^ bar^-1^) and the Nose–Hoover thermostat (310 K with three independent coupling groups for proteins, lipids, and water plus ions). Lennard-Jones and coulombic interactions were explicitly considered within a 12 Å cutoff, whereas the particle mesh Ewald method was used for electrostatic interactions beyond that distance (Darden et al., 1993). The system was first minimized with the steepest descent algorithm and was then equilibrated using a time step of 2 fs by applying positional restraints only to protein atoms (**Supplementary Table S3**), which were gradually released during 10 ns. Three replicas with different initial random velocities were initiated from the last snapshot of the equilibration and 100 ns were then performed in each case.

## Results

### Mapping of the TM Helices Involved in the A_2A_R–D_2_R Heterodimer Interface

Peptides corresponding to TM helices have been found to disrupt receptor–receptor interactions for GPCRs that form oligomers (Hebert et al., 1996; Ng et al., 1996; Borroto-Escuela et al., 2010b, 2012; Guitart et al., 2014; Lee et al., 2014; Jastrzebska et al., 2015; Vinals et al., 2015). The proposed mechanism of inhibition is that the helices are part of the protein–protein interface and are able to block oligomerization through competition with the other protomer. Structural information could thus be deduced by investigating the effects of the individual 14 TM helices of the two involved GPCRs on dimerization. In previous work, the ability of the TM helices of the D_2_R to disrupt A_2A_R–D_2_R heteromerization was evaluated by means of quantitative BRET^1^ assays (Borroto-Escuela et al., 2010b). The receptor–receptor interactions between the A_2A_R labeled with YFP (A_2A_R^Y FP^) and D_2_R fused to RLuc (D_2_R^RLuc^) were characterized for the inhibition of the BRET^1^ signal by the seven TM helices (TM-I^D2^ to TM-VII^D2^) in concentration-response experiments. Addition of synthetic peptides TM-IV^D2^ and TM-V^D2^ resulted in a clear concentration-dependent reduction of the BRET^1^ signal, leading to a nearly complete blockade at 1 to 10 μM. TM-VI^D2^ also achieved some inhibition at these concentrations, but not to the same extent as TM-IV^D2^ and TM-V^D2^. The remaining four peptides did not significantly influence A_2A_R–D_2_R heteromerization.

**FIGURE 1:**
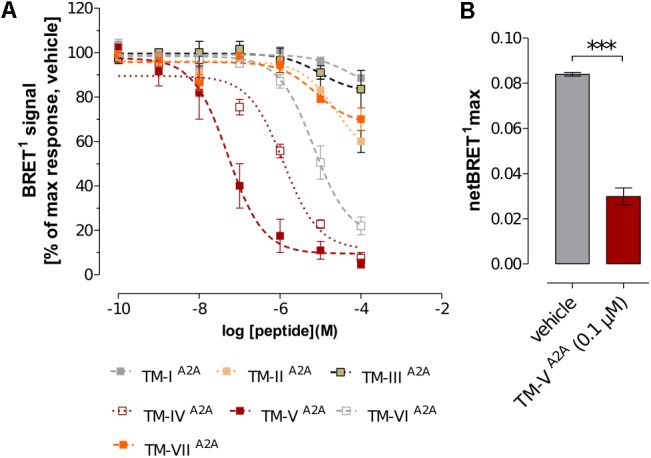
Effects of A_2A_R TM peptides on A_2A_R–D_2_R interactions. **(A)** The concentration-response effect of the individual A_2A_R TM peptides (TM-I^A2A^ to TM-VII^A2A^) on BRET^1^ signals of A_2A_R–D_2_R heteromers. Values represent percentages of maximal BRET^1^ responses in cells co-transfected with a 1:1 ratio of D_2_R^Rluc^ and A_2A_R^Y FP^ (1 μg cDNA each). Data are averages ± SEM; *n* = 6 experiments, 6 replicates (A_2A_R TM peptides I-III and V-VII); *n* = 6–10 experiments, 6 replicates (A_2A_R TM peptide IV). **(B)** Comparison of the effects of TM-V^A2A^ peptide with the vehicle group on the netBRET^1^ max values obtained from BRET^1^ saturation curves for the A_2A_R–D_2_R heteromers. Co-transfected cells were incubated with 0.1 μM of TM-V^A2A^ peptide or vehicle and studied by BRET^1^ saturation assays. Data are averages ± SEM; *n* = 4, eight replicates. ^∗∗∗^(TM-V^A2A^) significantly different compared to vehicle (*P* < 0.001) by unpaired *t*-test.

**FIGURE 2:**
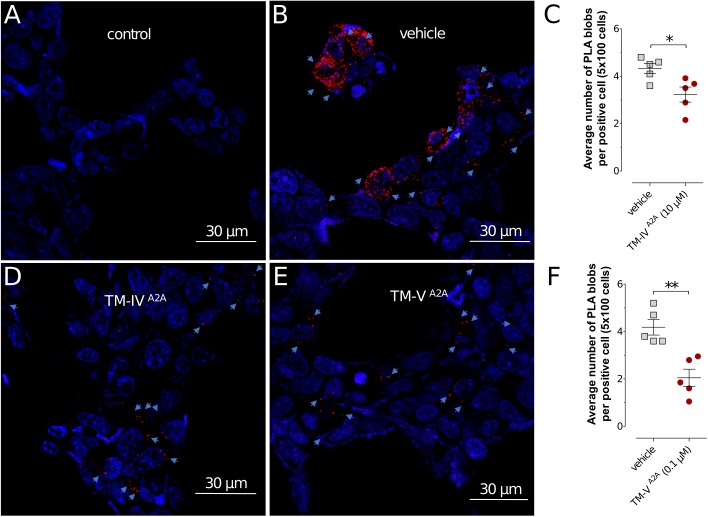
Detection of A_2A_R–D_2_R heteroreceptor complexes in transiently co-transfected HEK293T cells by *in situ* PLA. **(A,B,D,E)** The effects of A_2A_R TM peptides TM-IV^A2A^ (10 μM), TM-V^A2A^ (0.1 μM), and vehicle on the *in situ* PLA A_2A_R–D_2_R heteroreceptor complex signals are shown. Red clusters indicated by arrows represent heteroreceptor complexes and nuclei are shown in blue (DAPI). **(C,F)** Quantification of receptor complexes as the average number of PLA blobs (red clusters) per positive cell was determined using a confocal microscope Leica TCS-SL and the Duolink Image Tool software. Data are averages ± SEM (*n* = 5 experiments, 100 cells per experiment). Decreased A_2A_R–D_2_R heteroreceptor complex formation was observed upon cell incubation with TM-IV^A2A^ (10 μM) and TM-V^A2A^ (0.1 μM). These groups are significantly different compared to vehicle (^∗^*P* < 0.05; ^∗∗^*P* < 0.01). Statistical analysis was performed by unpaired *t*-test.

In the current work, the BRET^1^ experiments were performed for the seven TM peptides of the A_2A_R (TM-I^A2A^ to TM-VII^A2A^). TM-IV^A2A^, TM-V^A2A^, and TM-VI^A2A^ displayed a concentration-dependent inhibition of the BRET^1^ signal (**Figure 1A**). TM-V^A2A^ had the largest effect and A_2A_R–D_2_R dimerization was almost completely blocked at 1 μM. In a BRET saturation assay, TM-V^A2A^ clearly resulted in a significant reduction of the netBRET^1^max values at 0.1 μM (**Figure 1B**). TM-IV^A2A^ and TM-VI^A2A^ also reduced the BRET^1^ signal and reached close to full inhibition at 10 and 100 μM, respectively. TM-IV^A2A^ and TM-V^A2A^ were also evaluated by *in situ* PLAs to characterize the disruption of A_2A_R–D_2_R heteroreceptor complexes in transiently co-transfected HEK293T cells (TM-IV^A2A^, TM-V^A2A^) and in rat striatal primary neuronal cell culture (TM-V^A2A^). In line with the BRET^1^ assays, the *in situ* PLA assay showed that the number of clusters of A_2A_R–D_2_R heteroreceptor complexes was reduced upon addition of 10 μM of TM-IV^A2A^ and 0.1 μM TM-V^A2A^ (**Figure 2** and **Supplementary Figure S2**).

**FIGURE 3:**
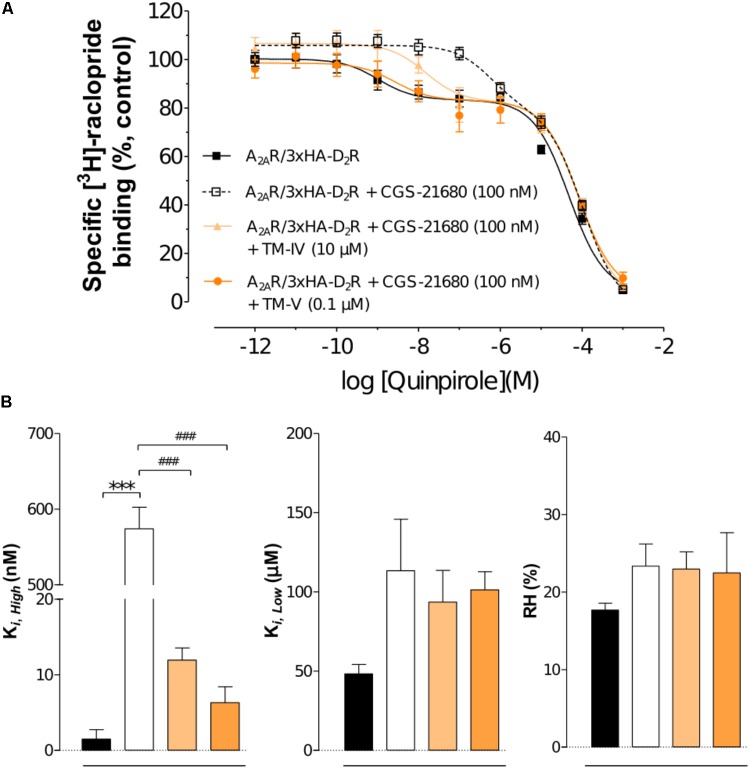
Experimental evaluation of TM peptides in radioligand binding assays. **(A)** Competition experiments involving D_2_R antagonist [^3^H]-raclopride binding versus increasing concentrations of quinpirole were performed in membrane preparation from HEK cells expressing A_2A_R–D_2_R in the presence of A_2A_R agonist CGS-21680 (100 nM), or A_2A_R agonist CGS-21680 (100 nM) + TM-IV^A2A^ (10 μM), or A_2A_R agonist CGS-21680 (100 nM) + TM-V^A2A^ (0.1 μM), or vehicle. Non-specific binding was defined as the binding in the presence of 10 μM (+)-butaclamol. The binding values (*n* = 3, in triplicate) are given in percent of specific binding at the lowest concentration of quinpirole employed. **(B)** Analysis and presentation of the high affinity values (K*_i,High_*), low affinity values (K*_i,Low_*), and the proportion of D_2_R in the high affinity state (RH). Averages ± SEM are given for three independent experiments performed in triplicate. Statistical analysis was performed by one-way ANOVA followed by Tukey’s Multiple Comparison test. ^∗∗∗^Significant difference compared to vehicle (*P* < 0.001). ^###^Significant difference compared to the CGS-21680 treatment (*P* < 0.001).

To evaluate the impact of the interfering peptides on the allosteric modulation within the A_2A_R–D_2_R heteromer, the affinity values for D_2_R agonist quinpirole for D_2_R were examined in [^3^H]-raclopride/quinpirole competition assays in HEK293T cells. The high affinity value (K*_i,High_*), the low affinity value (K*_i,Low_*), and the proportion of receptors in the high affinity state (RH) were not significantly altered when cells expressing only the D_2_R were treated with the TM-IV^A2A^ and TM-V^A2A^ peptides compared with the vehicle by one-way ANOVA (**Supplementary Figure S3**). The A_2A_R agonist CGS-21680 decreased the affinity of the high affinity component of the D_2_R agonist quinpirole in membrane preparations expressing A_2A_R–D_2_R, but the K*_i,Low_* and RH values were unaffected (**Figure 3**). Agonist binding to the high affinity state (K*_i,High_*) was reduced from 1.05 ± 0.36 nM to 583 ± 27 nM by the A_2A_ agonist CGS-21680. Thus, the strong negative allosteric modulation exerted by the A_2A_R agonist on the high affinity state of the wild type D_2_R was validated. In contrast, the allosteric modulation was significantly reduced in the experiments carried out in the presence of the TM-IV^A2A^ and TM-V^A2A^ peptides. TM-V^A2A^ resulted in the strongest effect and there was a complete loss of allosteric modulation (K*_i,High_* = 2.65 ± 0.52 nM). TM-IV^A2A^ also counteracted the effect on the high affinity component, resulting in a K*_i,High_* of 14.69 ± 0.35 nM. No differences in RH and K*_i,Low_* values were observed after the A_2A_R agonist modulation or treatment of TM peptides (**Figure 3**).

**FIGURE 4:**
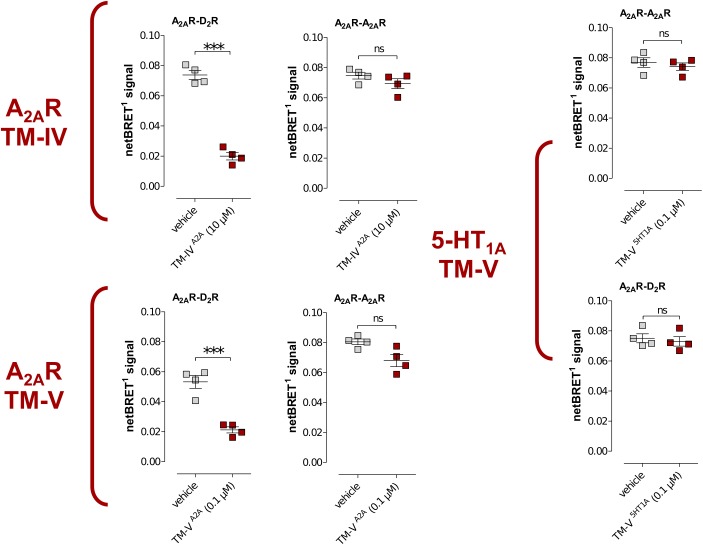
Effects of TM peptides on A_2A_R–D_2_R and A_2A_R–A_2A_R interactions. Cotransfected cells were incubated with either TM-IV^A2A^ (10 μM) or TM-V^A2A^ (0.1 μM) peptides or a peptide (0.1 μM) derived from TM-V of the serotonin 5-HT_1A_ receptor and studied by BRET^1^. Values represent netBRET responses (Averages ± SEM; *n* = 4, six replicates). Statistical analysis was performed by unpaired *t*-test. ^∗∗∗^*p* < 0.001 compared to control group.

To investigate the specificity of the A_2A_R TM peptides, BRET^1^ experiments were also carried out for the A_2A_R homodimer with a receptor population labeled with either YFP (A_2A_R^Y FP^) or Rluc (A_2A_R^RLuc^), which were compared to the results obtained for the A_2A_R–D_2_R heterodimer. Each peptide was assayed at a single-point concentration of 10 μM (TM-IV^A2A^) or 0.1 μM (TM-V^A2A^) and, interestingly, the results were different for the homo- and heterodimer. As expected, TM-IV^A2A^ and TM-V^A2A^ significantly reduced the population of A_2A_R–D_2_R complexes. However, no significant reduction of the BRET^1^ signal was observed for these two peptides in the case of A_2A_R homodimer. Similarly, the use of a peptide corresponding to TM-V of the serotonin 5-HT_1A_ receptor (Borroto-Escuela et al., 2012) did not have any significant effect on the A_2A_R–D_2_R heteromer or A_2A_R–A_2A_R homomer interactions (**Figure 4**).

### Experiment-Guided Modeling of the A_2A_R–D_2_R Heteroreceptor Complex

**FIGURE 5:**
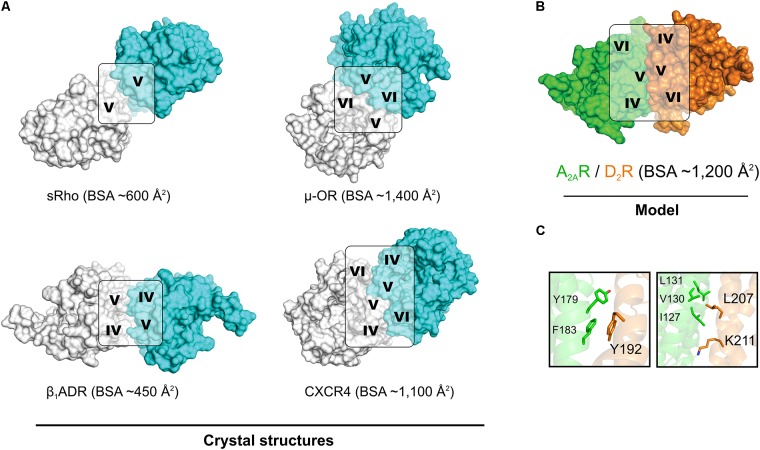
Crystal structures of GPCR homodimers with interfaces involving TM-IV, TM-V, and TM-VI and the model of the A_2A_R–D_2_R heterodimer. **(A)** Crystal structures of squid rhodopsin (sRho) (Murakami and Kouyama, 2008), μ-opioid (μ-OR) (Manglik et al., 2012), β_1_ adrenergic (β_1_ADR) (Huang et al., 2013), and CXCR4 (Wu et al., 2010) receptors. All these GPCRs crystallized as homodimers and have a large buried surface area (BSA). **(B)** Model of the heterodimer formed by the A_2A_R (green, crystal structure) and the D_2_R (orange, homology model). All structures are shown from the extracellular point of view and the TM helices involved in the dimerization interface are indicated. **(C)** Representative MD simulation snapshot of the mutated residues of the D_2_R (Top panel: Tyr192^5.41x42^, Bottom panel: Leu207^5.56^/Lys211^5.60^) with side chains of interacting residues shown as sticks.

Modeling of the A_2A_R–D_2_R complex was guided by the BRET^1^ data for the 14 TM peptides of the two protomers and dimer interactions observed in crystal structures of GPCRs. No crystal structures were available for heterodimers, but several class A GPCRs have been crystallized in homomeric arrangements, revealing different potential interfaces (**Figure 5A**) (Murakami and Kouyama, 2008; Wu et al., 2010; Granier et al., 2012; Manglik et al., 2012; Huang et al., 2013). The first cluster of observed dimer interfaces mainly involved TM-I, TM-II, and helix VIII and has, for example, been identified in crystal structures of the β_1_ adrenergic (Huang et al., 2013), κ- and μ-opioid (Granier et al., 2012; Manglik et al., 2012) receptors. As no effects of TM-I or TM-II on A_2A_R–D_2_R heteromerization were observed in the BRET^1^ assays, this interface was not further considered. A second set of interfaces involving TM-V and either TM-IV or TM-VI has been revealed by, e.g., crystal structures of squid rhodopsin (Murakami and Kouyama, 2008), CXCR4 (Wu et al., 2010), μ-opioid (Manglik et al., 2012), and β_1_ adrenergic (Huang et al., 2013) receptors. The synthetic peptides corresponding to the same three helices of the D_2_R and A_2A_R disrupted A_2A_R–D_2_R heterodimerization in the BRET^1^ assays (**Figure 1**) (Borroto-Escuela et al., 2010b). Visual inspection of the TM-IV/V/VI interfaces observed in the crystal structures of the CXCR4, μ-opioid, and β_1_ adrenergic receptors revealed that none of the observed dimers simultaneously involved all three helices to a significant extent. The μ-OR and squid rhodopsin receptor dimers had TM-V/VI and TM-V interfaces, respectively, but not TM-IV, which showed the largest effect among the synthetic peptides derived from the D_2_R. The β_1_ adrenergic receptor had a TM-IV/V interface, but the buried surface area (BSA) was relatively small (450 Å^2^). The CXCR4 receptor structure had strong interactions via TM-V and also involved TM-IV and TM-VI to a smaller extent, resulting in the largest BSA (∼1,100 Å^2^).

In order to generate a model of the A_2A_R–D_2_R heterodimer, atomic resolution structures for the monomeric forms of each protomer were required. Multiple crystal structures of the A_2A_R in monomeric form were available and the one with the highest resolution, which represented an inactive state conformation, was selected (Liu et al., 2012). At the initiation of this study, no crystal structure of the D_2_R was available. Thus, a crystal structure of the D_3_R (Chien et al., 2010) was used to generate a set of homology models of the D_2_R in an inactive-like state. Due to the high sequence similarity to the D_3_R, which shared 71% of the amino acids in the TM region with the D_2_ subtype (**Supplementary Figure S1**), there was only a small variation among the models generated. The three best models, as judged by DOPE score (Shen and Sali, 2006), were analyzed manually and one of these was selected as a starting point for modeling of the heterodimer. Models of the A_2A_R–D_2_R complex were first generated by structural superimposition of the monomers onto the available homodimer crystal structures with interfaces that involved TM-IV and TM-V. CXCR4 (Wu et al., 2010) was considered to be the best template for the A_2A_R–D_2_R heterodimer based on the large contact interface involving TM-V, but the resulting model only had weak interactions with TM-IV. Protein–protein docking with the program HADDOCK (Dominguez et al., 2003) was performed to identify interfaces that were in better agreement with the experimental data for the TM peptides. HADDOCK allows inclusion of experimental data to focus the docking calculation on a specific interface. Residues in TM-IV and TM-V were defined as being involved in the interface based on the BRET data and CXCR4-based model (**Supplementary Table S2**). The protein–protein docking was used to generate 200 models of the A_2A_R–D_2_R complex, which were clustered based on RMSD. The 10 most populated clusters are shown in **Supplementary Figure S4** and were analyzed based on four criteria (**Supplementary Table S4**). The first two criteria required the receptors to be oriented in a parallel manner and correctly aligned with the plane of the membrane-solvent interface, which was fulfilled by 133 models from four different clusters. Among these, the heterodimer models in the sixth cluster were found to have the most extensive contacts involving TM-IV and TM-V and its centroid was selected for further consideration (**Figure 5B** and **Supplementary Figure S5**). The predicted A_2A_R–D_2_R interface had a total BSA of ∼1,200 Å^2^ and was overall similar, but not identical, to the model obtained by aligning to the CXCR4 homodimer crystal structure (**Supplementary Figure S6**). TM-V was the helix with the highest contribution to the dimer interface for both protomers. However, the selected structure had some rearrangements compared to CXCR4-based model that led to higher involvement of TM-IV.

### MD Simulation Refinement of the A_2A_R–D_2_R Heterodimer Model

The A_2A_R–D_2_R heterodimer predicted by protein–protein docking was further explored using all-atom MD simulations in a hydrated lipid bilayer. The time-scales reachable by MD simulations are too short to observe major rearrangements of the dimer interface, structural changes related to activation of the receptors, or the influence of oligomerization on ligand binding. Therefore the goal of these calculations was to refine the predicted interface using a more realistic model of the biological environment. Three independent trajectories of the dimer model were generated, resulting in a total of 0.3 μs of unrestrained simulation. After a short equilibration (**Supplementary Table S3**), the overall root mean square deviation (RMSD) of the Cα atoms of the dimer increased slightly during the first ∼40 ns of unrestrained simulation and then stabilized at an average of 3.3 Å, which is similar to the results obtained for a simulation study on the crystal structure of the CXCR4 homodimer (Rodriguez and Gutierrez-De-Teran, 2012). The largest structural changes were observed in TM-VII of the A_2A_R and TM-I of the D_2_R whereas the dimer interface remained stable. The BSA changed only slightly during the simulation, with an average of 1,163 Å^2^ for the last 50 ns of the three simulation replicas (**Supplementary Figure S7** and **Supplementary Table S5**).

Snapshots from the MD simulations were analyzed to identify interactions between specific residues in the interface. To further evaluate the model, two D_2_R mutants that probed interactions in either the N- or C-terminal ends of these helices were predicted (**Figure 5C**). The interaction interface at the top of TM-V was close to the orthosteric binding sites of both receptors. Tyr192^5.41x42^ of the D_2_R was located just one helical turn from a set of residues (Ser193^5.42x43^, Ser194^5.43x44^, and Ser197^5.46x461^) that has been proposed to play a role in the activation mechanism for monoamine-recognizing GPCRs (Warne et al., 2011). The side chain of Tyr192^5.41x42^ formed stacking interactions with a cluster of aromatic residues formed by Tyr179^5.40x411^ and Phe183^5.44x45^ in TM-V of the A_2A_R, which were located only ∼10 Å from the adenosine binding site. At the intracellular end of TM-V of the D_2_R, interactions with residues in TM-IV and TM-V of the A_2A_R were formed. Leu207^5.56^ of the D_2_R interacted with Ile127^4.48^, Val130^5.51^, and Leu131^4.52^ of the A_2A_R whereas the alkyl chain of Lys211^5.60^ formed van der Waals interactions with Ile127^4.48^ of the A_2A_R. Based on these results, two mutants of the D_2_R, Tyr192Ala^5.41x42^ and Leu207Ala^5.56^/Lys211Ala^5.60^, were predicted to reduce favorable dimer interactions and were selected for experimental evaluation.

### Experimental Evaluation of D_2_R Mutations in the Predicted Heteromer Interface

**FIGURE 6:**
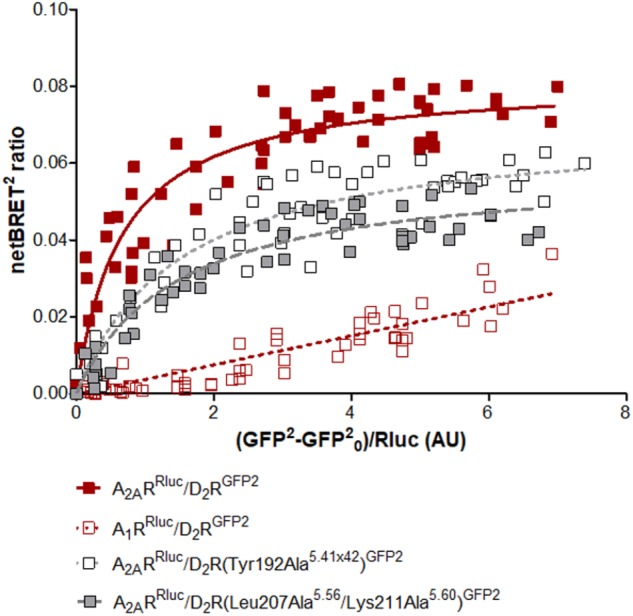
Experimental evaluation of mutations in the A_2A_R–D_2_R dimer interface by BRET^2^. BRET^2^ saturation curves for the A_2A_R–D_2_R heterodimer for wild type and mutants of the D_2_R. The normalized BRET^2^ ratio, which was defined as the netBRET^2^ ratio for co-expressed Rluc and GFP^2^ constructs normalized against the netBRET^2^ ratio for the Rluc expression construct alone in the same experiment, is plotted on the *y*-axis. The fluorescence value obtained from the GFP^2^, normalized with the luminescence value of A_2A_R^Rluc^ or A_1_R^Rluc^ expression 10 min after *DeepBlueC* incubation, is plotted on the *x*-axis. GFP_0_ is defined as the fluorescent emission values at 515 ± 30 nm of the cells which only expressed the Rluc construct. Data are averages ± SEM; *n* = 5, eight replicates. Statistical analysis was performed by unpaired *t*-test. The netBRETmax values of A_2A_R–D_2_R(Tyr192Ala^5.41x42^) are significantly different compared to A_2A_R–D_2_R (*P* < 0.05) and the netBRETmax values of A_2A_R–D_2_R(Leu207Ala^5.56^/Lys211Ala^5.60^) are significantly different compared to A_2A_R–D_2_R (*P* < 0.01).

**FIGURE 7:**
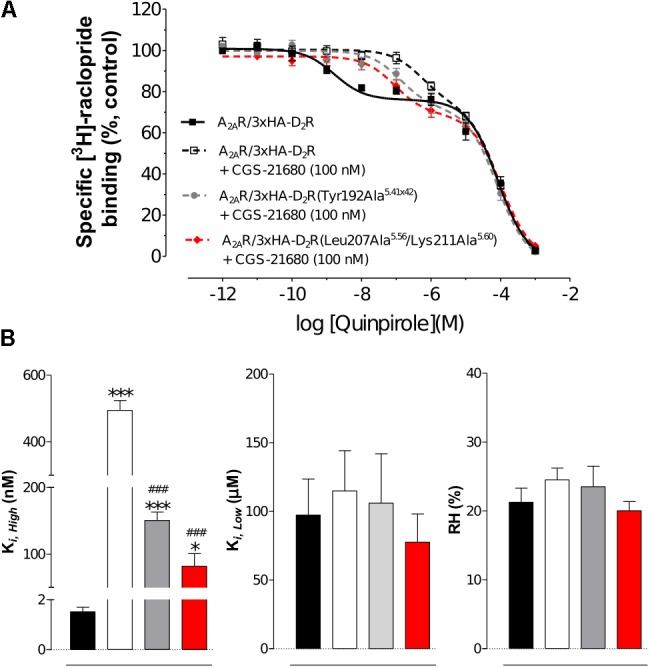
Experimental evaluation of mutations in the A_2A_R–D_2_R dimer interface by radioligand binding assays. **(A)** Competition experiments involving D_2_R antagonist [^3^H]-raclopride binding versus increasing concentrations of quinpirole were performed in membrane preparation from HEK cells expressing A_2A_R/3xHA-D_2_R or A_2A_R/3xHA-D_2_R(T192A^5.41x42^), or A_2A_R/3x-HA-D_2_R(L207A^5.56^/K211A^5.60^) in the presence or absence of A_2A_R agonist CGS-21680 (100 nM). Non-specific binding was defined as the binding in the presence of 10 μM (+)-butaclamol. The binding values (*n* = 4, in triplicate) are given in percent of specific binding at the lowest concentration of quinpirole employed. **(B)** Analysis and presentation of the CGS-21680 induced changes in the high affinity values (K*_i,High_*), low affinity values (K*_i,Low_*) and in the proportion of D_2_R in the high affinity state (RH) after incubation of the membrane preparations with A_2A_R agonist CGS-21680 (100 nM). Data are averages ± SEM of four independent experiments, each one performed in triplicate. Statistical analysis was performed by one-way ANOVA followed by Tukey’s Multiple Comparison test. ^∗∗∗^*P* < 0.001 and ^∗^*P* < 0.05; Significant difference compared to wild-type without CGS-21680. ^###^*P* < 0.001; The results for the two mutants were significantly different compared to wild-type with CGS-21680.

To further probe the predicted role of TM-V of the D_2_R in A_2A_R–D_2_R heteromerization, the Tyr192Ala^5.41x42^ and Leu207Ala^5.56^/Lys211Ala^5.60^ D_2_R mutants were evaluated experimentally in BRET^2^, *in situ* PLA, and binding assays. No differences in cellular localization at the membrane level between the wild type and mutants of the D_2_R were observed (**Supplementary Figure S8**). The interactions between the wild type and mutant D_2_Rs labeled with GFP2 with the A_2A_R^RLuc^ were first assessed in BRET^2^ saturation assays. Both the Tyr192Ala^5.41x42^ and Leu207Ala^5.56^/Lys211Ala^5.60^ mutations reduced A_2A_R–D_2_R interactions and the largest effect was observed for the double mutant (*p* < 0.01, **Figure 6**). In contrast, no significant differences were observed between the wild type and mutant D_2_Rs with the A_2A_R protomer if the *in situ* PLA method was used (**Supplementary Figure S9**). In binding assays, the high affinity value (K*_i,High_*) for D_2_R agonist quinpirole for 3xHA-D_2_R, 3xHA-D_2_R(Tyr192Ala^5.41x42^), and 3xHA-D_2_R(Leu207Ala^5.56^/Lys211Ala^5.60^) were determined in [^3^H]-raclopride/quinpirole competition assays (1.65 ± 0.21 nM, 1.08 ± 0.34 nM, and 1.80 ± 0.22 nM, respectively). The high affinity value (K*_i,High_*), the low affinity value (K*_i,Low_*) and the proportion of receptors in the high affinity state (RH) were not significantly altered for the two D_2_R mutants compared with the wild type D_2_R by one-way ANOVA (**Supplementary Figure S10**). As expected, the A_2A_R agonist CGS-21680 decreased the affinity of the high affinity component of the D_2_R agonist quinpirole in membrane preparations expressing A_2A_R/3xHA-D_2_R whereas the K*_i,Low_* and RH values were not significantly altered. The D_2_R agonist binding to the high affinity state was reduced 300-fold by the A_2A_ receptor agonist CGS-21680 to 533 ± 23 nM. Significant A_2A_R agonist induced reductions of the K*_i,High_* value for quinpirole were also observed for the D_2_R mutants, but not to the same degree as found in the wild type. The Tyr192Ala^5.41x42^ and Leu207Ala^5.56^/Lys211Ala^5.60^ mutants had (K*_i,High_*) values equal to 129 ± 21 nM and 90 ± 2 nM in the presence of A_2A_R agonist, respectively. The ability of 3xHA-D_2_R(Tyr192Ala^5.41x42^) and 3xHA-D_2_R(Leu207Ala^5.56^/Lys211Ala^5.60^) to respond to the negative allosteric modulation induced by the A_2A_R agonist was hence significantly lower than for the wild type A_2A_R/3xHA-D_2_R complex. No significant differences in RH and K_i,Low_ values were observed (**Figure 7**).

### Model of the A_2A_R–D_2_R Complex Based on Crystal Structures of Both Protomers

**FIGURE 8:**
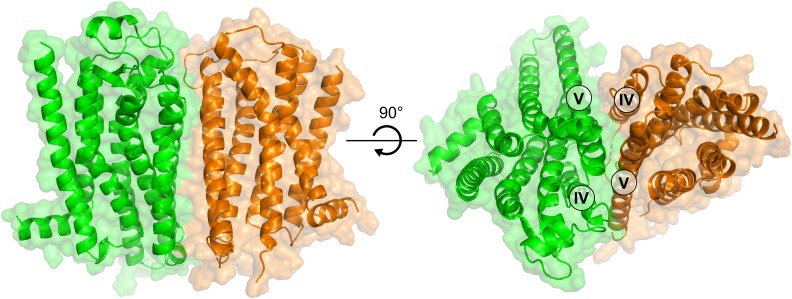
Model of the A_2A_R–D_2_R heterodimer based on crystal structures of both receptors. The structure of the A_2A_R–D_2_R heteroreceptor complex is based on protein–protein docking with the A_2A_R (green) and D_2_R (orange) depicted as cartoons.

After the initial review of this paper, the first crystal structure of the D_2_R was determined (Wang et al., 2018). This allowed us to assess the accuracy of the D_2_R model and generate alternative interfaces of the A_2A_R–D_2_R dimer using protein–protein docking based on crystal structures of both receptors. Alignment of the D_2_R homology model and crystal structure showed that the D_3_R subtype was a good template for the prediction of the TM region (backbone RMSD = 1.2 Å). To assess the influence of using a D_2_R homology model on our results and to further improve our model of the A_2A_R–D_2_R complex, the protein–protein docking calculations were carried out with the crystal structures. A total of 1,000 models were generated and the resulting clusters of potential interfaces were assessed using the same criteria as for the D_2_R homology model (**Supplementary Table S6**). The third most populated cluster was compatible with membrane insertion and had a TM-IV/V interface. The interface RMSD between the cluster center obtained from docking with the D_2_R homology model (cluster 6, **Supplementary Table S4**) and crystal structure (cluster 3, **Supplementary Table S6**) was 4.6 Å. These models hence belonged to the same cluster of solutions (HADDOCK clustering cut-off < 7.5 Å) and the interfaces were very similar based on our criteria (**Supplementary Figure S11**). On the residue level, there were some differences in the interactions predicted by the two cluster centers, but the contacts made by the residues evaluated by mutagenesis (Tyr192Ala^5.41x42^ and Leu207Ala^5.56^/Lys211Ala^5.60^) were maintained in the A_2A_R–D_2_R complex obtained using the crystal structures (**Supplementary Figure S11**). A representative A_2A_R–D_2_R model obtained using crystal structures of both receptors is shown in **Figure 8**.

## Discussion

The main result of this study is that identification of dimer disrupting peptides derived from TM helices in combination with crystal structure data for GPCRs enabled the generation of atomic-resolution models of the A_2A_R–D_2_R heteroreceptor complex. A representative A_2A_R–D_2_R model was refined using MD simulations and mutations in the predicted interface reduced the propensity of the receptors to dimerize.

A key step toward understanding GPCR heteromerization is the identification of the regions involved in receptor–receptor interactions (Meng et al., 2014; Cordomi et al., 2015). In the membrane-spanning region, essentially every TM helix has been proposed as a potential interface (Selent and Kaczor, 2011; Ferre et al., 2014). For example, in the case of the A_2A_R–D_2_R heteromer, Canals et al. (2003) proposed that the TM interface was mainly composed of TM-V/VI/VII (D_2_R) and TM-III/IV (A_2A_R) whereas the study by Borroto-Escuela et al. (2010b) predicted that TM-IV/V (D_2_R) interacted with either TM-IV/V (A_2A_R) or TM-I/VII (A_2A_R). In the current work, the interface of the A_2A_R–D_2_R heteromer was further characterized by probing the ability of peptides based on the TM helices of the A_2A_R to interfere with receptor–receptor interactions. The TM-IV and TM-V peptides had the strongest effect on A_2A_R–D_2_R interactions. Combined with the corresponding data for the D_2_R (Borroto-Escuela et al., 2010b), the results pointed to a primary interface involving TM-IV/V for both the A_2A_R and D_2_R, but may also involve interactions with TM-VI. This model is in agreement with the work of Canals et al. (2003), in which a chimera of the D_1_- and D_2_R subtypes was characterized. To probe the role of the TM region of the D_2_R in heteromerization, Canals et al. (2003) replaced TM-V/VI and IL3 with the corresponding sequence of the D_1_ subtype, which does not dimerize with the A_2A_R. The D_2_/D_1_ chimera did not interact with the A_2A_R, supporting a TM interface involving either TM-V, TM-VI, IL3 or a combination of these regions. These results are consistent with our experimental data for peptides corresponding to TM-V of the D_2_R, which resulted in a reduction of heteromerization in BRET assays (Borroto-Escuela et al., 2010b). As TM-IV, TM-V, and TM-VI cannot be part of a single interface, TM-IV/V interactions were prioritized over those involving TM-V/VI based on the fact that the TM-VI peptide resulted in the smallest effects experimentally. In addition, the fact that the degree of A_2A_R–D_2_R heteromerization is not affected by receptor activation (Canals et al., 2003) makes an TM-V/VI interface less likely because this complex would not allow the large conformational changes in TM-VI required for G protein binding (Rasmussen et al., 2011b). An alternative interpretation is that the dimer interface changes upon activation, as proposed for the D_2_R (Guo et al., 2005) and metabotropic glutamate receptor 2 homodimers (Xue et al., 2015). Although this is a more complex model, it is possible that there is an equilibrium between the TM-IV/V and TM-V/VI interfaces, which may be influenced by activation of either protomer. The results for the TM peptides and predicted interactions are also in agreement with the recent findings by Navarro et al. (2018), which also identified TM-IV/V as the primary interface of the A_2A_R–D_2_R heterodimer.

With a more detailed map of the helices involved in A_2A_R–D_2_R heteromerization, specific residue interactions and their role in allosteric modulation of ligand binding can be probed. Previous studies have investigated the role of intracellular regions in A_2A_R–D_2_R heteromerization by site directed mutagenesis. Based on pulldown and mass spectrometry experiments, negatively charged residues in the C-terminal tail of the A_2A_R were proposed to form strong interactions with an arginine rich region in IL3 of the D_2_R (Ciruela et al., 2004). Mutagenesis of the set of arginine residues in IL3 of the D_2_R (217–222 and 267–269) to alanine abolished the negative allosteric modulation of D_2_R agonist on activation of the A_2A_R (Fernandez-Duenas et al., 2012). Similarly, site-directed mutagenesis for three residues in the C-terminal tail of the A_2A_R (Ser374, Asp401, and Asp402) supported a role of this intracellular region in heteromerization. Loss of heteromerization for the alanine mutant of phosphorylated Ser374, the double mutant Asp401Ala/Asp402Ala as well as the combination of these three mutations were demonstrated in BRET assays. The two mutants involving Ser374 also disrupted the negative allosteric modulation mediated by the A_2A_R on high-affinity agonist binding to the D_2_R (Borroto-Escuela et al., 2010a). The C-terminal of the A_2A_R and IL3 of the D_2_R were not included in our proposed heterodimer model because of the lack of reliable templates to predict these regions. However, it should be noted that the positively charged region in IL3 of the D_2_R is close to our proposed TM-IV/V interface as these residues are located at the intracellular part of TM-V. Considering that the three negatively charged residues of the A_2A_R are located at the end of the long C-terminal tail, interactions between these two regions are also compatible with our proposed model.

To further test the predicted TM interface of the A_2A_R–D_2_R complex, two D_2_R variants with mutations in either the N- or C-terminal end of TM-V were evaluated experimentally. None of these mutations disrupted dimerization completely, which was expected considering the large predicted contact surface and the involvement of intracellular regions in receptor–receptor interactions. The BRET data indicated a reduced population of heteroreceptor complexes, which supports the view that TM-V of the D_2_R is part of the interface. The lack of effect in the PLA experiments may be due to a combination of the relatively modest impact of the mutants on dimerization and high receptor expression, which can lead to saturation effects that influence quantification of small differences in the level of receptor–receptor interactions using this technique (Mocanu et al., 2011). Based on the binding data, the predicted TM-IV/V interactions between the two receptors could contribute to the negative allosteric modulation across the A_2A_R–D_2_R heterodimer. Interestingly, crystal structures of the A_2A_R in active- and inactive-like conformations suggest that TM-V shifts slightly inward upon activation by adenosine (Lebon et al., 2011). A similar inward contraction of TM-V was observed for monoamine-recognizing GPCRs that are homologous to the D_2_R (e.g., for the β_1_ and β_2_ adrenergic receptors) (Rasmussen et al., 2011a; Warne et al., 2011). The negative allosteric modulation across the A_2A_R–D_2_R heterodimer may thus be partly accomplished via interactions across the TM-V interface. The reduction of agonist binding to the D_2_R observed upon activation of the A_2A_R may be due to altered helix–helix interactions when TM-V shifts inward in response to adenosine receptor activation. In the complex with an A_2A_R antagonist, TM-V can instead contribute to stabilizing the high affinity state conformation of the D_2_R binding site, leading to the increased dopaminergic D_2_R signaling sought in treatment of Parkinson’s disease (Fuxe et al., 2007b; Armentero et al., 2011).

The A_2A_R and D_2_R exist both as homo- and heteromers in the brain and alteration of the balance between these receptor complexes influences intracellular signaling and could lead to development of neurological diseases (Fuxe and Borroto-Escuela, 2016). The heteromer interface between the A_2A_R and D_2_R was the main focus of this work. Future studies need to consider potential changes to the interface upon receptor activation, the influence of homodimers on A_2A_R–D_2_R interactions, and the existence of tetramers composed by A_2A_R–A_2A_R and D_2_R–D_2_R complexes (Bonaventura et al., 2015). As a first step toward characterizing the A_2A_R homodimer, the effects of peptides derived from TM-IV and TM-V of the A_2A_R on this complex were evaluated experimentally. In contrast to the strong inhibition of dimerization observed with these peptides for the A_2A_R–D_2_R heteromer, there were no effects on A_2A_R homomerization. The TM-IV^A2A^ and TM-V^A2A^ peptides hence selectively modulate the A_2A_R–D_2_R heteromer, which supports the view that the A_2A_R homo- and A_2A_R–D_2_R heteromerization involve different helices.

In this work, we developed an approach for mapping the interfaces of GPCR dimers based on biophysical experiments in combination with protein–protein docking and MD simulations. The modeling of the A_2A_R–D_2_R heterodimer was guided by information from low-resolution experiments that identified helices involved in receptor–receptor interactions and led to a potential TM-IV/V interface. The proposed A_2A_R–D_2_R structure provides a starting-point for designing new experiments that can contribute to further refinement of the model and understanding of allosteric modulation at the molecular level. The same approach can likely be extended to the large number of class A GPCRs that have been shown to engage in receptor–receptor interactions.

## Author Contributions

DR, JK, MJ, AR, and JC designed, performed, analyzed, and interpreted all the molecular modeling studies. DB-E, WR-F, TL, and KF designed, performed, analyzed, and interpreted all laboratory experiments. All authors contributed to the writing of the manuscript.

## Conflict of Interest Statement

The authors declare that the research was conducted in the absence of any commercial or financial relationships that could be construed as a potential conflict of interest.
