# Supplementary material for: Mapping the Interface of a GPCR Dimer: A Structural Model of the A2A Adenosine and D2 Dopamine Receptor Heteromer
Source: Front Pharmacol. 2018 Aug 30;9:829. doi: 10.3389/fphar.2018.00829 (PMC6125358; doi:10.3389/fphar.2018.00829)
Supplement: Supplementary file 1 [file Data_Sheet_1.pdf]

Supplementary Material

Mapping the interface of a GPCR dimer: A structural model of the A_{2A} adenosine and D_2 dopamine receptor heteromer

Dasiel O. Borroto-Escuela, David Rodriguez, Wilber Romero-Fernandez, Jon Kapla, Mariama Jaiteh, Anirudh Ranganathan, Tzvetana Lazarova, Kjell Fuxe* and Jens Carlsson*

***Correspondence:**

Jens Carlsson: jens.carlsson@icm.uu.se

Kjell Fuxe: kjell.fuxe@ki.se

1 Supplementary Figures and Tables

1.1 Supplementary Figures

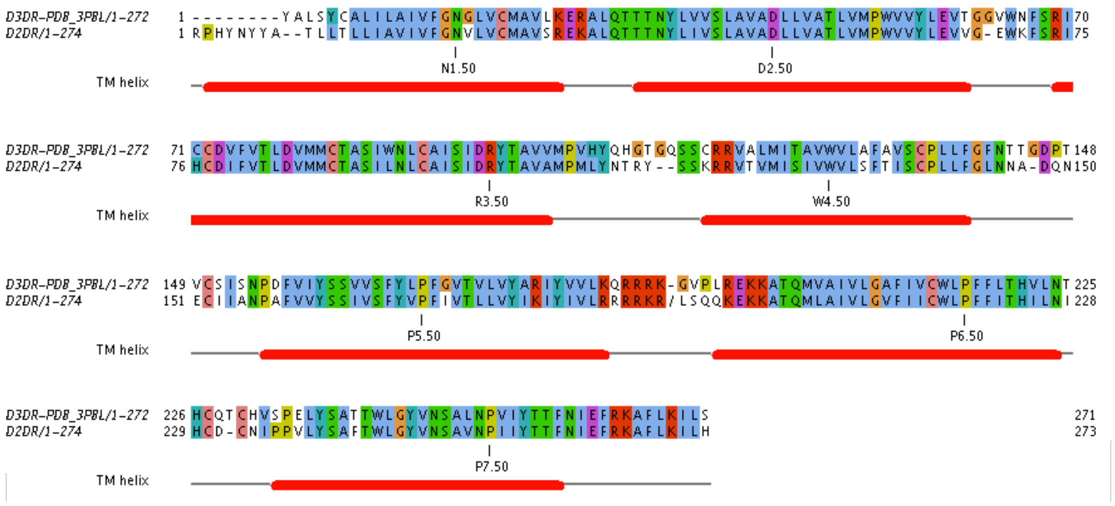

Figure S1. Sequence alignment used to generate the D_2R homology model. A crystal structure of the D_3R subtype was used to generate homology models of the D_2R. Labels indicate the location of the TM helices and the most conserved residue for each of them across class A GPCRs.

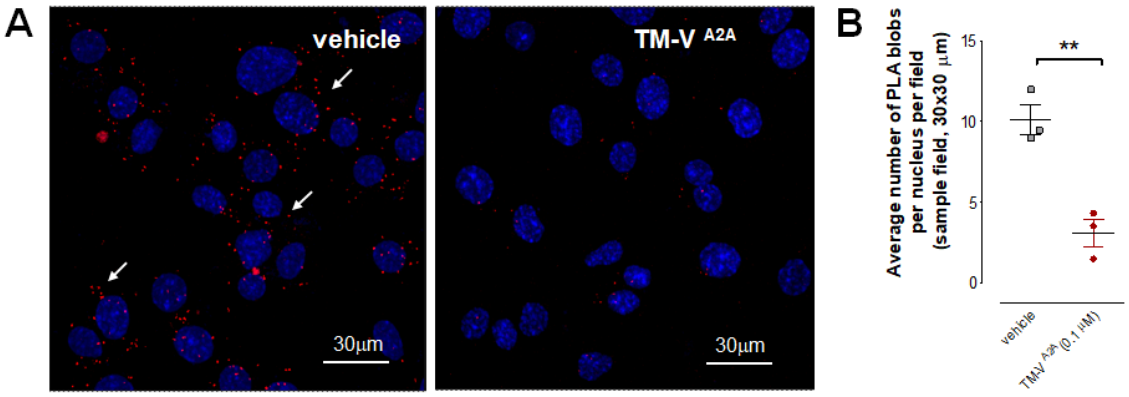

Figure S2. Detection of $A_{2A}R$-D_2R heteroreceptor complexes in rat primary striatal neuronal cells by *in situ* PLA. Detection of $A_{2A}R$-D_2R heteroreceptor complexes was carried out with both primary antibodies (rabbit polyclonal anti-$A_{2A}R$ (Millipore, AB1559)) and the mouse monoclonal anti-D_2R (Millipore MABN53, clone 3D9) in rat primary striatal neuronal cells. (A) The effects of TM-V^{A2A} (0.1 μM) and vehicle on the *in situ* PLA $A_{2A}R$-D_2R heteroreceptor complex signals are shown. Red clusters, indicated by arrows, represent heteroreceptor complexes and nuclei are shown in blue (DAPI). (B) Quantification of receptor complexes as red clusters per DAPI-positive nucleus per sampled field (30x30 μm) was determined using a confocal microscope Leica TCS-SL and the Duolink Image Tool software. Data are averages ± SEM (n=3 experiments, 40 pictures in each experiment). Decreased $A_{2A}R$-D_2R heteroreceptor complex formation was observed upon cell incubation with TM-V^{A2A} (0.1 μM). This group is significantly different compared to vehicle (**: P<0.01). Statistical analysis was performed by unpaired t-test.

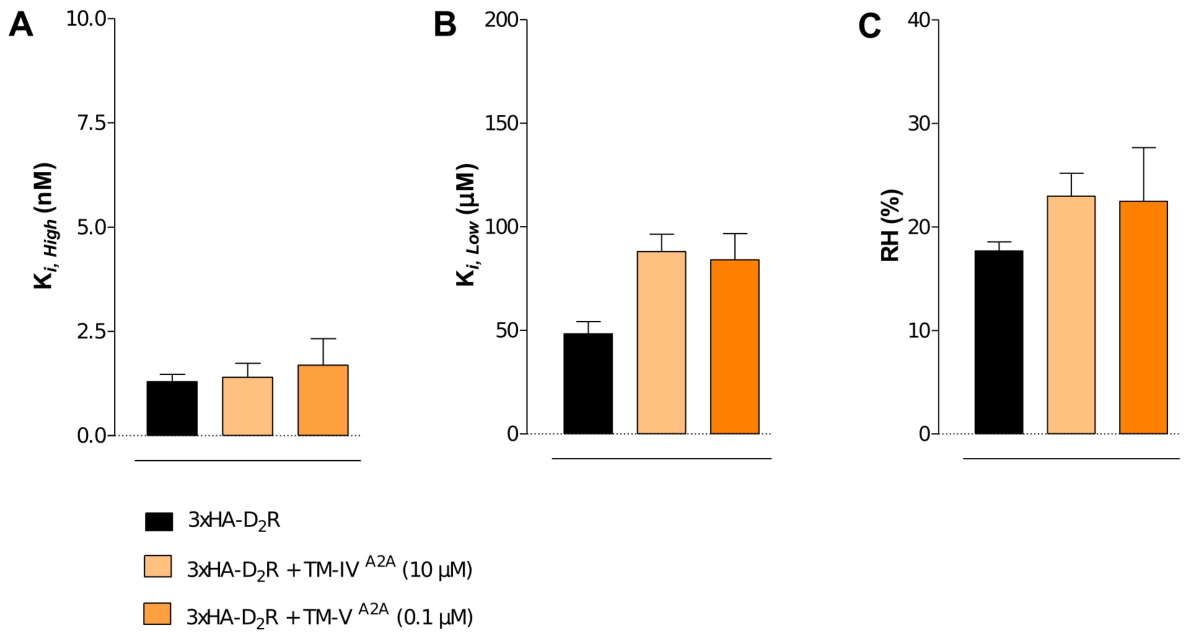

Figure S3. Effect of $A_{2A}R$ transmembrane peptides on D_2R receptor ligand binding. The effects of TM-IV^{A2A} (10 µM), TM-V^{A2A} (0.1 µM), and vehicle on the competition experiments involving D_2R antagonist [^{3}H]-raclopride binding versus increasing concentrations of quinpirole in membrane preparation from HEK cells expressing only the D_2R are shown. Nonspecific binding was defined as the binding in the presence of 10 µM (+)-butaclamol. (A) High affinity values ($K_{i,High}$), (B) low affinity values ($K_{i,Low}$), and (C) the proportion of D_2R in the high affinity state (RH). Data are averages ± SEM for 3 independent experiments, each one performed in triplicate. Statistical analysis was performed by one-way ANOVA followed by Tukey's Multiple Comparison test.

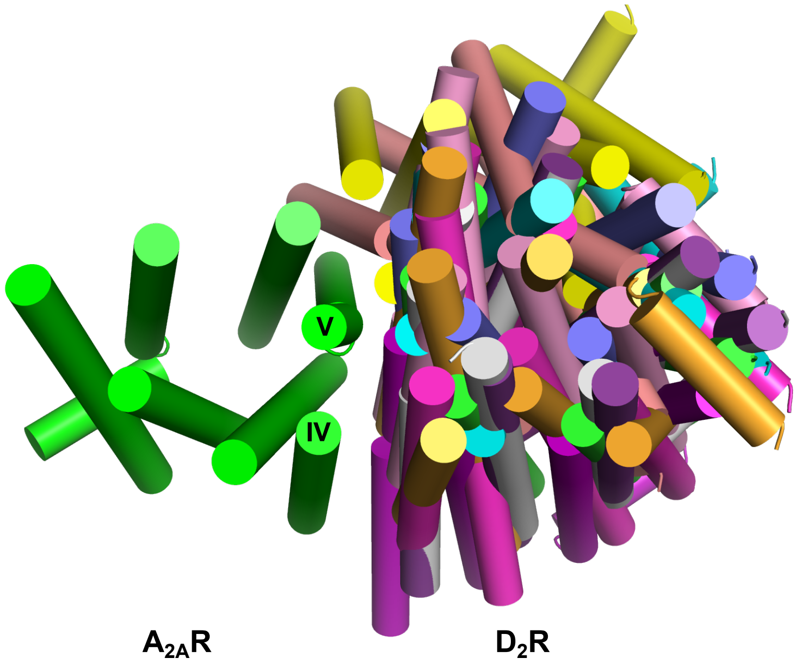

Figure S4. Results from protein-protein docking. The ten largest clusters from the protein-protein docking (represented by cluster centers) with restraints that focus sampling on a TM-IV/V interface. The receptor helices are shown as cylinders and for the D_2R each color represents a different cluster center. Loop regions have been omitted for clarity.

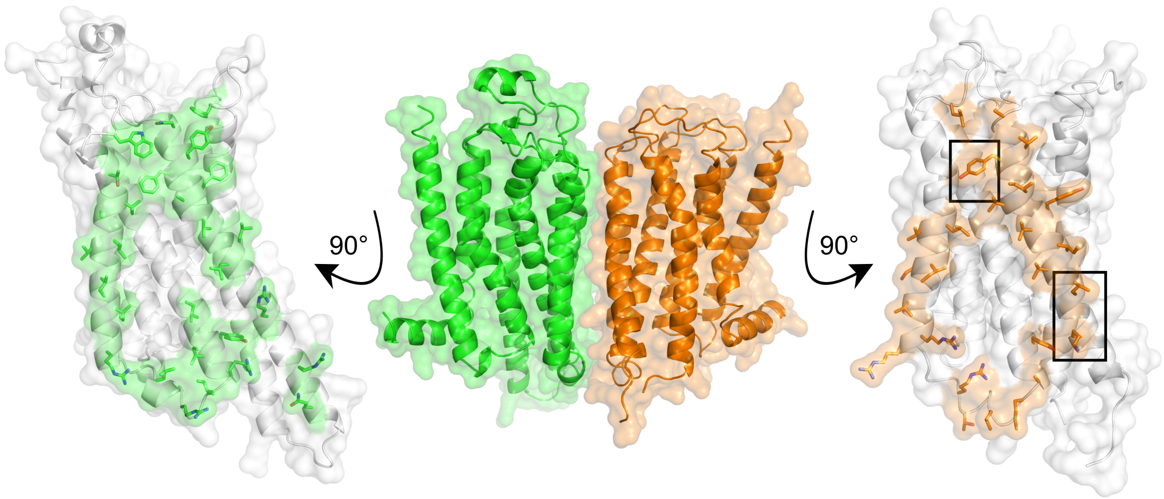

Figure S5. Model of the $A_{2A}R$-D_2R heterodimer. Model of the $A_{2A}R$-D_2R heteroreceptor complex (middle panel) based on a crystal structure of the $A_{2A}R$ (green) and homology model of the D_2R (orange) depicted as cartoons with key residues in sticks. The dimer interfaces of the $A_{2A}R$ (left panel) and D_2R (right panel) are shown as surfaces. The residues selected for the mutagenesis study are indicated with black rectangles.

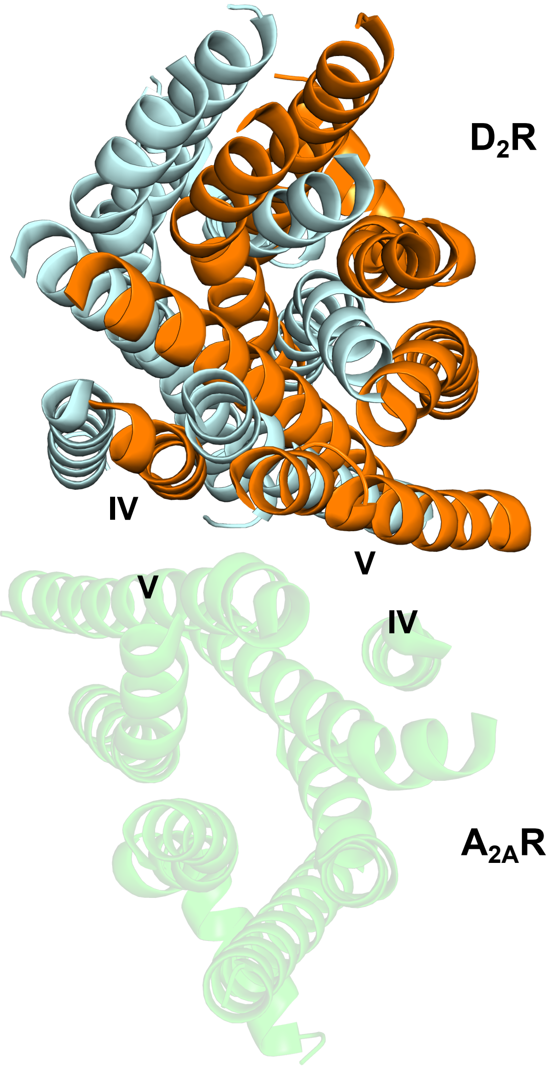

Figure S6. Comparison of $A_{2A}R$-D_2R models obtained from alignment to a CXCR4 homodimer and protein-protein docking. The receptors are shown as cartoons and the $A_{2A}R$ is colored green. The models of the heterodimer that were obtained by alignment to the CXCR4 crystal structure and by protein-protein docking are shown in blue and orange, respectively. Loop regions have been omitted for clarity.

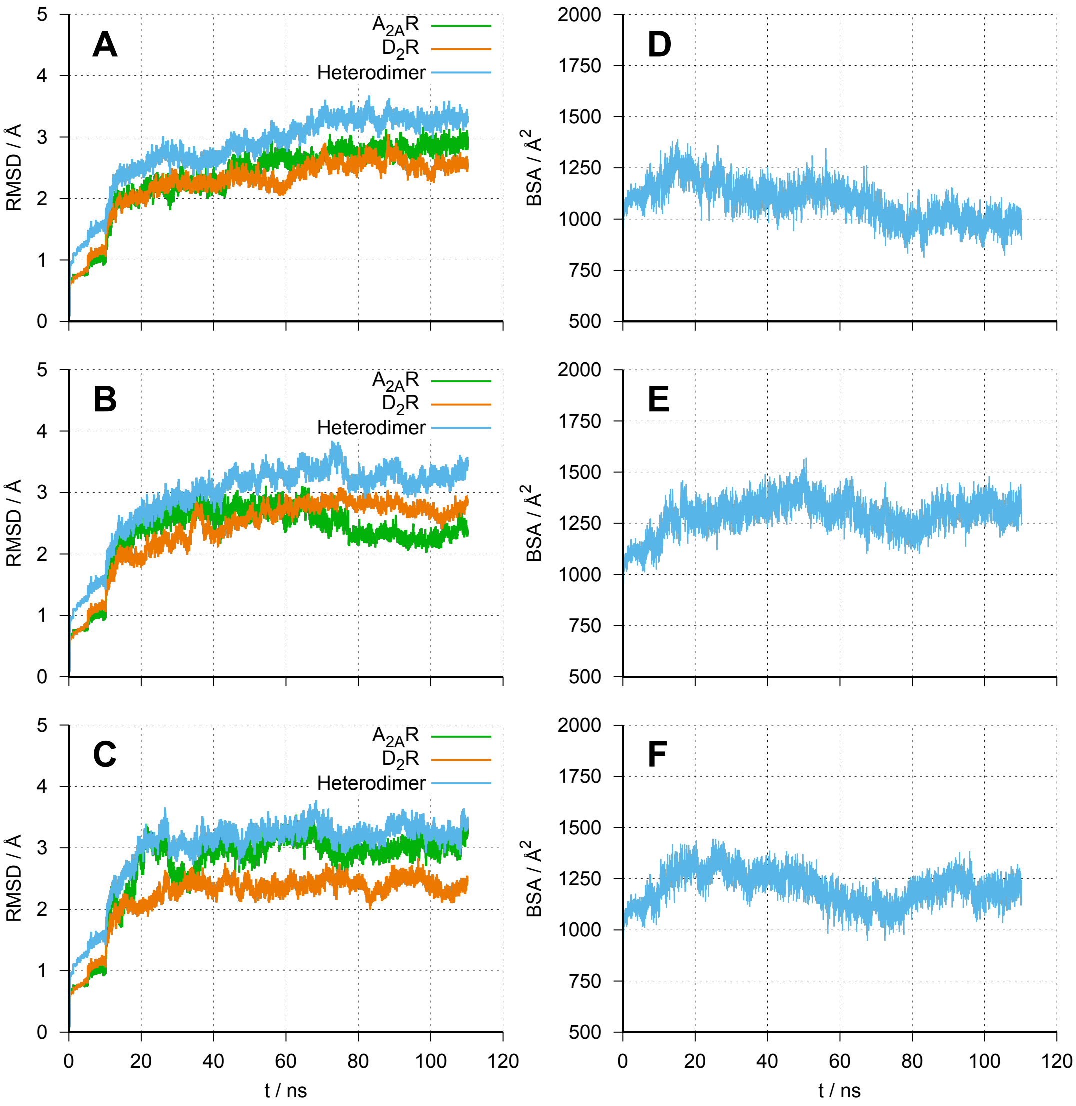

Figure S7. Root-mean-squared deviation (Cα RMSD) for snapshots from MD simulations to the model generated by protein-protein docking and buried surface area (BSA) for trajectories S1 (A, D), S2 (B, E), and S3 (C, F).

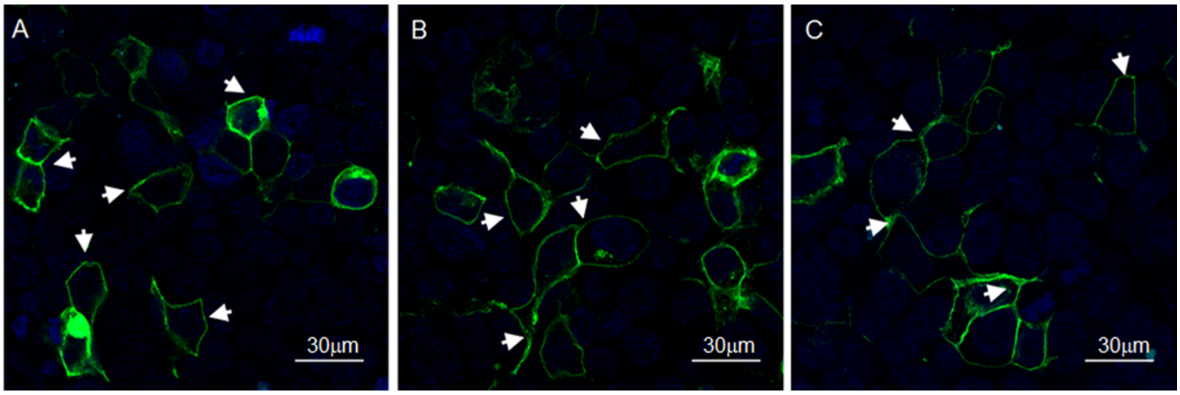

Figure S8. Cellular localization of D_2R wild type and mutants in HEK293T cells. Fluorescence confocal microscopy was used to visualize (green) the localization of wild type and mutants of the D_2R 48 hours after transient transfection. In cells expressing (A) 3xHA-D_2R^{GFP2} (control), (B) 3xHA-D_2R^{GFP2}(Tyr192Ala$^{5.41x42}$), and (C) 3xHA-D_2R^{GFP2}(Leu207Ala$^{5.56}$/Lys211Ala$^{5.60}$), the receptors mainly localized to the cell membrane (see arrows). Nuclei are shown in blue (DAPI). The images are shown as a single z-scan image and are representative of three individual experiments.

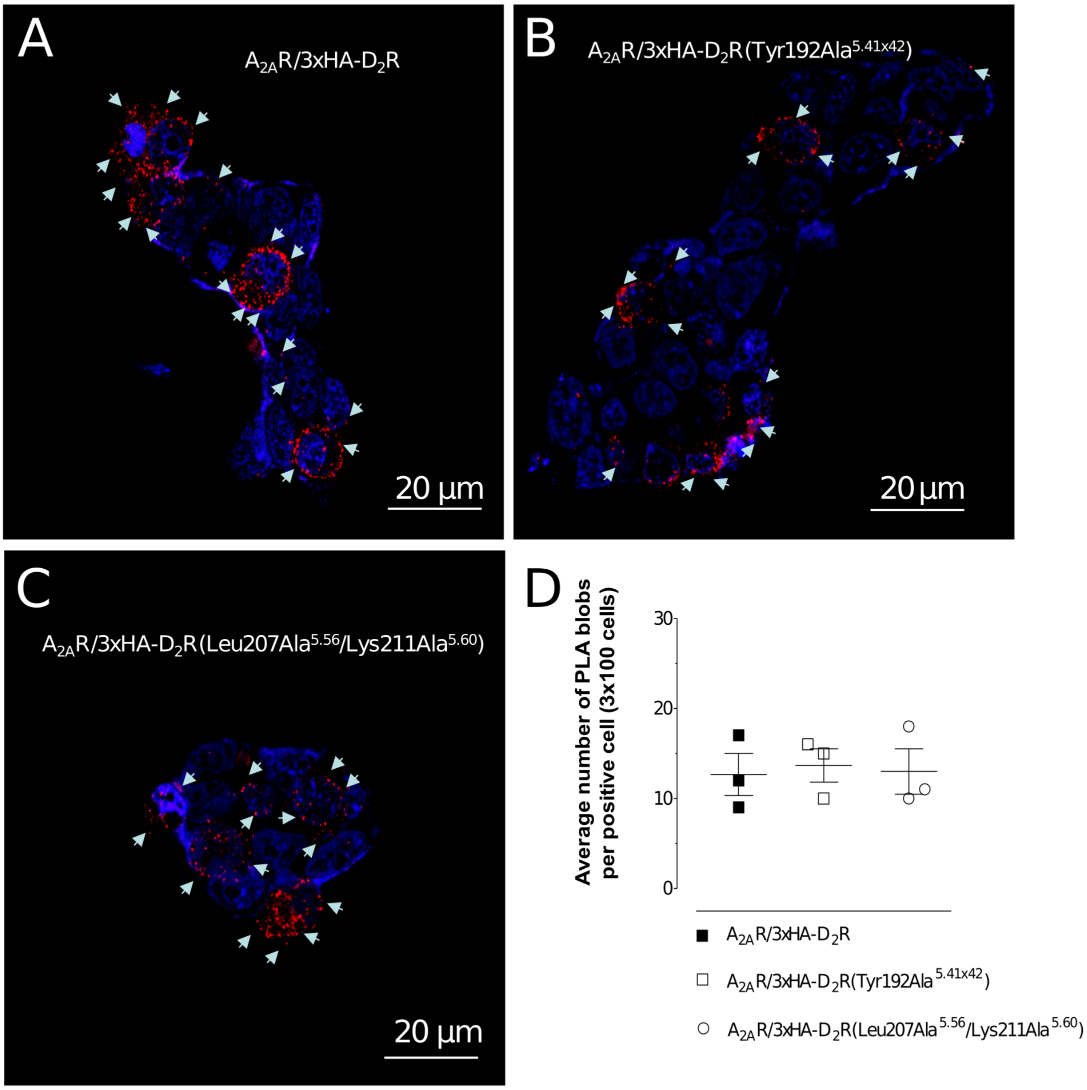

Figure S9. Detection of $A_{2A}R$-D_2R heteroreceptor complexes in co-transfected HEK293T cells by *in situ* PLA. Detection of $A_{2A}R$-D_2R heteroreceptor complexes was carried out with both primary antibodies (rabbit polyclonal anti-$A_{2A}R$ (Millipore, AB1559)) and the mouse monoclonal anti-D_2R (Millipore MABN53, clone 3D9) in cells expressing (A) $A_{2A}R$/3xHA-D_2R, (B) $A_{2A}R$/3xHA-D_2R(Tyr192Ala$^{5.41x42}$), or (C) $A_{2A}R$/3xHA-D_2R(Leu207Ala$^{5.56}$/Lys211Ala$^{5.60}$). The effects of D_2R mutants and vehicle on the *in situ* PLA $A_{2A}R$-D_2R heteroreceptor complex signals are shown. Red clusters indicated by arrows represent heteroreceptor complexes and nuclei are shown in blue (DAPI). (D) Quantification of receptor complexes as the average number of PLA blobs (red clusters) per positive cell was determined using a confocal microscope Leica TCS-SL and the Duolink Image Tool software. Data are averages ± SEM (n=3 experiments, 100 cells per experiment). No significant $A_{2A}R$-D_2R heteroreceptor complex formation was observed by one-way ANOVA followed by Tukey's Multiple Comparison test.

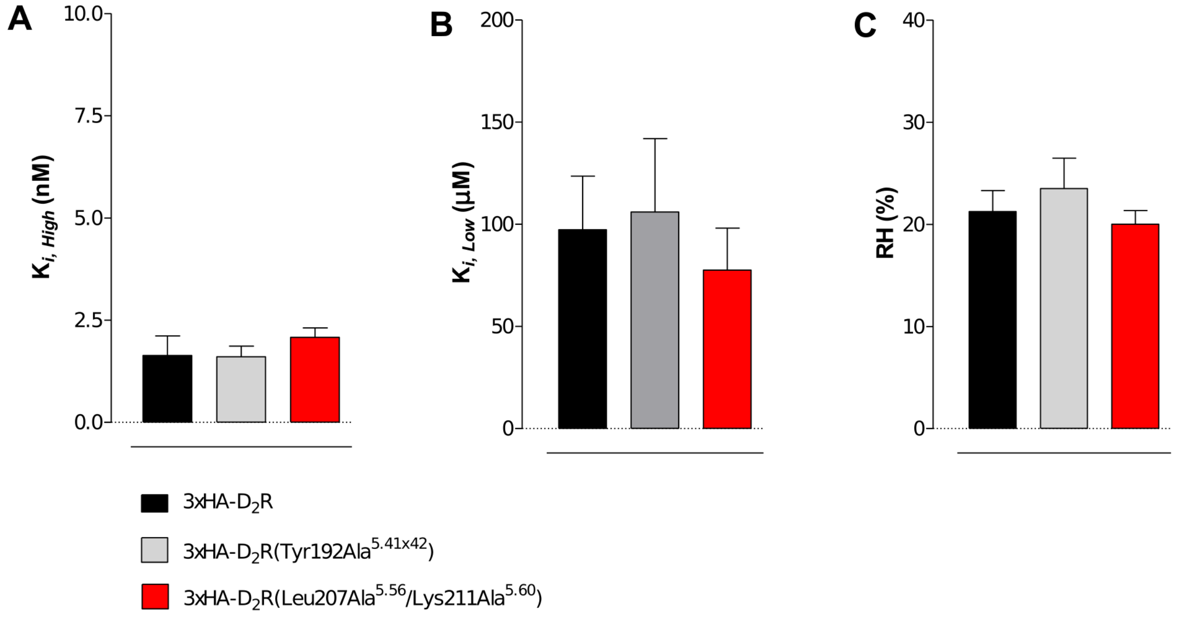

Figure S10. Effect of D_2R mutations on ligand binding. Competition experiments involving D_2R antagonist [^{3}H]-raclopride binding versus increasing concentrations of quinpirole were performed in membrane preparation from HEK cells expressing 3xHA-D_2R or 3xHA-D_2R(Tyr192Ala$^{5.41x42}$) or 3x-HA-D_2R(Leu207Ala$^{5.56}$/Lys211Ala$^{5.60}$). Nonspecific binding was defined as the binding in the presence of 10 µM (+)-butaclamol. (A) High affinity values ($K_{i,High}$), (B) low affinity values ($K_{i,Low}$), and (C) the proportion of D_2R in the high affinity state (RH). Data are averages ± SEM for 4 independent experiments, each one performed in triplicate. Statistical analysis was performed by one-way ANOVA followed by Tukey's Multiple Comparison test.

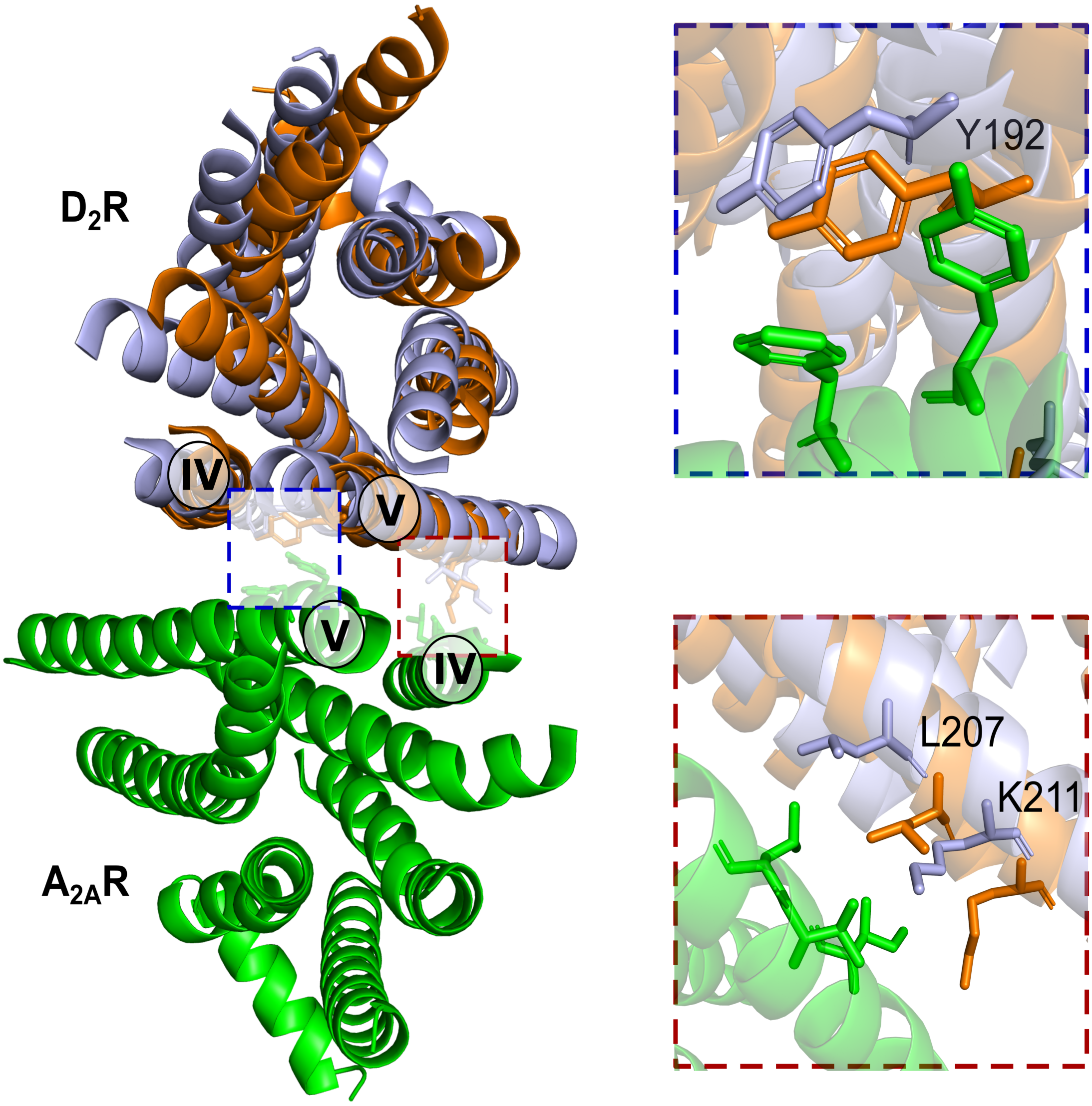

Figure S11. Comparison of $A_{2A}R$-D_2R complexes obtained from protein-protein docking with a homology model and crystal structure of the D_2R. The receptors are shown as cartoons and the $A_{2A}R$ is colored green. The models of the heterodimer that were obtained with the D_2R homology model and crystal structure are shown in orange and purple, respectively. The TM backbone RMSD between the D_2R model and crystal structure is 5.4 Å.

Supplementary Tables

Table S1. Protonation states of histidine residues in the model of the $A_{2A}R$-D_2R heterodimer.

Receptor	Residue	Protonation state
$A_{2A}R$	$His75^{3.33}$	Protonated in Nε
	$His155^{EL2}$	Protonated in Nε
	$His230^{6.32}$	Protonated in Nε
	$His250^{6.52}$	Protonated in Nε
	$His264^{EL3}$	Charged
	$His278^{7.43x42}$	Protonated in Nδ
	$His306^{8.61}$	Protonated in Nε
D_2R	$His106^{3.24}$	Protonated in Nε
	$His393^{6.55}$	Protonated in Nε
	$His398^{6.60}$	Protonated in Nε
	$His442^{8.59}$	Protonated in Nε

Table S2. Residues used to define restraints for protein-protein docking. See methods section for further details.

Ambiguous restraints[a]

**A$_{2A}$R**		**D$_2$R**	
**Active residues**	**Passive residues**	**Active residues**	**Passive residues**
Ile100$^{3.48}$	Gly123$^{4.44}$	Thr134$^{3.52}$	Thr144$^{34.55}$
Arg107$^{3.55}$	Ile124$^{4.45}$	Ala137$^{3.55}$	Ser157$^{4.47}$
Pro109$^{34.50}$	Asn175$^{5.36x38}$	Met138$^{3.56}$	Val161$^{4.51}$
Leu110$^{34.51}$	Leu194$^{5.55}$	Leu141$^{34.52}$	Leu162$^{4.52}$
Arg111$^{34.52}$	Gly195$^{5.56}$	Arg145$^{34.56}$	Tyr199$^{5.48}$
Leu115$^{34.56}$	Leu198$^{5.59}$	Arg150$^{4.40}$	Pro201$^{5.50}$
Arg120$^{4.41}$	Arg205$^{5.66}$	Arg151$^{4.41}$	Ile210$^{5.59}$
Tyr179$^{5.40x411}$	Ser213$^{5.74}$	Val154$^{4.44}$	
Ala184$^{5.45x46}$	Asn253$^{6.55}$	Ile158$^{4.48}$	
Val188$^{5.49}$	Pro260$^{EL3(6.62)}$	Pro187$^{5.36x37}$	
Leu191$^{5.52}$		Ala188$^{5.37x38}$	
Leu192$^{5.53}$		Val191$^{5.40x41}$	
Arg199$^{5.60}$		Ile195$^{5.44x45}$	
Leu202$^{5.63}$		Val200$^{5.49}$	
Arg206$^{5.67}$		Ile203$^{5.52}$	
Lys209$^{5.70}$		Leu207$^{5.56}$	
Gln210$^{5.71}$		Lys211$^{5.60}$	
Phe258$^{6.60}$		Ile214$^{5.63}$	
		Arg218$^{5.67}$	
		Arg220$^{5.69}$	
		His398$^{6.60}$	

Unambiguous restraints[b]

**A$_{2A}$R**	**D$_2$R**
Tyr179$^{5.40x411}$	Ala188$^{5.37x38}$

[a]Ambiguous restraints were defined for one set of residues and these were either classified as "active", which belonged to the core of the dimer interface, or "passive", which were solvent-accessible residues adjacent to the "active" ones.

[b]An unambiguous restraint between residues in the extracellular part of the receptors was introduced to enrich dimer solutions compatible with their parallel arrangement in the membrane.

Table S3. Equilibration scheme for the $A_{2A}R$-D_2R dimer in all-atom MD simulations. The force constant used to restrain the positions of different protein atom subsets was gradually reduced during a 10 ns equilibration scheme. The rest of the system (lipids, waters, and ions) was unrestrained during equilibration.

**Step**	**Atoms**	**Force constant (kJ mol^{-1} nm^{-2})**	**Length (ns)**
1	Protein (non-hydrogen) heavy atoms	1000	1
2		800	1
3		600	1
4		400	1
5		200	1
6	Protein Cα atoms	200	5

Table S4. Analysis of the 10 clusters of predicted $A_{2A}R$-D_2R structures obtained from protein-protein docking using an $A_{2A}R$ crystal structure and D_2R homology model.

Cluster	Members[a]	Parallel orientation[b]	Membrane aligned[c]	TM-IV interactions[d]	TM-V interactions[e]
**1**	91	YES	YES	NO	YES
**2**	23	YES	YES	NO	YES
**3**	22	NO	NO	YES	YES
**4**	12	YES	NO	NO	YES
**5**	11	YES	NO	YES	YES
**6**	10	YES	YES	YES	YES
**7**	9	YES	YES	NO	YES
**8**	6	NO	NO	YES	YES
**9**	5	YES	NO	YES	YES
**10**	5	NO	NO	YES	YES

[a] The number of members in the cluster. Each cluster was required to have at least four members. All solutions in the same cluster have a pair-wise interface backbone RMSD < 7.5 Å.

[b] The receptors are oriented with the extracellular and intracellular regions pointing in the same direction.

[c] Both receptors in the dimer model are aligned with the expected planes of the membrane. Predicted orientations of the receptors in the membrane were obtained from the OPM database (http://opm.phar.umich.edu/)

[d] TM-IV of one protomer is interacting with the other protomer.

[e] TM-V of one protomer is interacting with the other protomer.

Table S5. Root-mean-squared deviation (RMSD, average ± standard deviation) and buried surface area (BSA, average ± standard deviation) for three MD simulation trajectories (S1-3) of the $A_{2A}R$-D_2R heterodimer. The averages are calculated over the second half of the trajectories.

		**RMSD (Å)**	**BSA (Å^2)**
**S1**	Heterodimer	3.2 ± 0.1	1026 ± 67
	$A_{2A}R$	2.8 ± 0.1	
	D_2R	2.5 ± 0.2	
**S2**	Heterodimer	3.3 ± 0.1	1295 ± 61
	$A_{2A}R$	2.4 ± 0.2	
	D_2R	2.8 ± 0.1	
**S3**	Heterodimer	3.3 ± 0.1	1168 ± 63
	$A_{2A}R$	3.0 ± 0.1	
	D_2R	2.4 ± 0.1	

Table S6. Analysis of the 10 clusters of predicted $A_{2A}R$-D_2R structures obtained from protein-protein docking using crystal structures of both protomers.

Cluster	Members[a]	Parallel orientation[b]	Membrane aligned[c]	TM-IV interactions[d]	TM-V interactions[e]
**1**	415	YES	NO	YES	YES
**2**	197	YES	YES	NO	YES
**3**	151	YES	YES	YES	YES
**4**	92	NO	NO	YES	YES
**5**	90	YES	YES	NO	YES
**6**	11	YES	NO	YES	YES
**7**	8	YES	NO	YES	YES
**8**	8	NO	NO	NO	YES
**9**	4	NO	NO	YES	YES
**10**	4	NO	NO	YES	YES

[a] The number of members in the cluster. Each cluster was required to have at least four members. All solutions in the same cluster have a pair-wise interface backbone RMSD < 7.5 Å.

[b] The receptors are oriented with the extracellular and intracellular regions pointing in the same direction.

[c] Both receptors in the dimer model are aligned with the expected planes of the membrane. Predicted orientations of the receptors in the membrane were obtained from the OPM database (http://opm.phar.umich.edu/)

[d] TM-IV of one protomer is interacting with the other protomer.

[e] TM-V of one protomer is interacting with the other protomer.
